# Remanufacturing Strategy Choice of a Closed-Loop Supply Chain Network Considering Carbon Emission Trading, Green Innovation, and Green Consumers

**DOI:** 10.3390/ijerph19116782

**Published:** 2022-06-01

**Authors:** Yan Zhou, Xin-Tong Lin, Zhi-Ping Fan, Kar-Hung Wong

**Affiliations:** 1 Department of Management Science and Engineering, Qingdao University, Qingdao 266071, China; yanzhou@qdu.edu.cn (Y.Z.); halted_lin@163.com (X.-T.L.); 2 School of Business Administration, Northeastern University, Shenyang 110169, China; 3 School of Computer Science and Applied Mathematics, University of the Witwatersrand, Johannesburg 2000, South Africa; karwong01@gmail.com

**Keywords:** closed-loop supply chain, remanufacturing strategy choice, carbon emission trading, green innovation, green consumers, circular economy

## Abstract

Due to increasingly serious environmental pollution problems and governments’ strengthening of environmental impact supervision, manufacturing companies are seeking green production methods, implementing carbon trading systems, and promoting the trend towards green remanufacturing. Thus, this paper introduces green factors to the existing closed-loop supply chain network models and studies the impact of carbon trading, green innovation efforts, and green consumers on the choice between two remanufacturing strategies: an in-house remanufacturing strategy and an authorized remanufacturing strategy. The results concerning the choice of the remanufacturing strategy are as follows: from the perspective of obtaining more profits, when the carbon trading price is low, the companies choose the authorized remanufacturing strategy; when the carbon trading price is high, the companies choose to remanufacture by themselves. For all the green innovation efforts and the proportions of green consumers, when the recovery rate of the used product is low, the companies choose to remanufacture by themselves; when the recovery rate of the used product is high, the companies choose the authorized remanufacturing strategy.

## 1. Introduction

Due to the rapid development of industrialization, resource shortages are becoming a serious problem. To achieve sustainable development, enterprises throughout the world are actively seeking different methods to combat this problem, and remanufacturing is one of these methods. Thus, people are seeking various means to employ techniques to improve the qualities of products and increase their lifespan via the collection and repair of used products [1]. Many countries have enacted legislation to promote the collection and remanufacturing of waste products [2]. Remanufacturing can bring more benefits to manufacturing companies; for example, Bosch collected used products and recycled them to increase its profits [3]; Kodak used discarded cameras for recycling and remanufacturing and encouraged the partners in the supply chain to support recycling to obtain an additional income [4]. Remanufacturing enables enterprises to make full use of their resources to achieve maximum benefits [5,6,7].

On the other hand, due to the worsening of the global atmosphere and the degradation of the environment, the realization of green development is important in another way. Countries have adopted various measures to promote carbon trading and effective policies to reduce carbon emissions [8], and green innovation is the basis for realizing a “win-win” scenario of economic growth and environmental protection [9]. Consequently, more companies have begun to invent various methods to improve companies’ performance in carbon trading, which develops the abilities and the competitive spirits among all the employees in the supply chain for trading green products [10].

In the other direction of study, the optimal management of a closed-loop supply chain (CLSC), which involves competition in a supply chain cycle of an enterprise from procurement to final sales, has attracted the attention of many researchers. Basically, the CLSC network is a network structure which comprises multiple manufacturers, retailers, and remanufacturers competing in a separate contrasting relationship. The literature in the study of the CLSC network [11,12,13,14,15] assumes that the new products and remanufactured products in the product flow are the same in quality and price, but different consumers have different product preferences, and consumers will choose new products or remanufactured products according to their self-interest and the qualities of the good. To cope with the preferences of the consumers, Apple has carried out research on consumers’ demands for new or remanufactured products before it considers producing new phones [16]. Therefore, this paper also separates the remanufactured products from the new products in the CLSC network so that the demands for these products can be substantially studied.

The existing research on the CLSC network only considers the original equipment manufacturer (OEM) in-house remanufacturing model; it does not consider the authorized strategy and does not compare the performances between the in-house remanufacturing and the authorized remanufacturing. In the actual production process, the method to obtain a license for remanufacturing has become the most significant issue for remanufacturers. In foreign countries, the ink cartridge remanufacturers of Canon and Epson faced the risk of being litigated by Lexmark for remanufacturing without its permission [17]. To study these issues in great depth, this paper focuses on the equilibrium of the CLSC network considering the OEM’s in-house remanufacturing and authorized remanufacturing strategy choices. Moreover, the demand for green products from green consumers can prompt companies to make green innovations. Efforts to carry out green production, and green innovation efforts can help manufacturing companies improve their competitiveness. The differences between the new and remanufactured products together with other factors, such as carbon emission trading, green innovation effort, and green consumers, should also play a critical role in the choice of remanufacturing strategies. Therefore, this paper studies the influence of these green factors on the choice between the two remanufacturing strategies, and the research on carbon emission trading, green innovation, and green consumers in this paper can provide companies with more realistic decision-making plans, which will help them to actively adopt strategy changes, i.e., in-house remanufacturing and authorized remanufacturing, and contribute to the renewal of the companies by finding a greener production method for remanufacturing. More precisely, based on the equilibrium between different factors such as carbon emission trading, green innovation, and green consumers, this paper guides the OEM on how to choose a strategy between in-house remanufacturing and authorizing a third-party remanufacturer (3PR) for remanufacturing, i.e., authorized remanufacturing, according to profit and environmental impact. The research results provide enterprises with more choices concerning remanufacturing strategies and guides the enterprises through their exploration of a more profitable production mode.

The contributions of this article are as follows:

(1) Unlike the current research about the CLSC network equilibrium problem, this paper provides two product flows in the forward product flow direction so that consumers can choose between new and remanufactured products based on their green preferences;
(2) This paper establishes an equilibrium model of the CLSC networks under the two different remanufacturing strategies, i.e., in-house remanufacturing and authorized remanufacturing, and thus we obtain the equilibrium of the CLSC network and obtain a method for selecting between the above mentioned two remanufacturing strategies;
(3) This paper incorporated green factors, such as the carbon trading system, green innovation effort, and the proportion of green consumers to non-green consumers, into the existing CLSC network models to help decision-makers to choose between the strategies of using in-house remanufacturing or the authorized remanufacturing strategy. Moreover, by changing the values of the parameters of different green factors, we can obtain the boundary conditions of the remanufacturing strategy choices.

The organization of the remainder of this paper is as follows: the literature review is discussed in the next section, the models of two remanufacturing strategies are given in Section 3, Section 4 and Section 5, and the numerical analysis and the conclusion are given in Section 6 and Section 7, respectively.

## 2. Literature Review

### 2.1. Remanufacturing Strategy

Remanufacturing engineering is designed to repair the used products in such a way that the qualities of the remanufactured products can meet or exceed those of new products. Recent studies have shown that product remanufacturing can increase the demand for remanufactured products, affect the production quantities of new products, and further enable the manufacturer and the whole supply chain to obtain better production methods from the manufacturer recycling model [5,6,7,17]. The main processing method for remanufacturing products is called authorized remanufacturing, in which the original equipment manufacturer (OEM) is only responsible for selling new products; after charging third party remanufacturers (3PR) an authorization fee, the recycling, remanufacturing, and sale of the remanufactured products are authorized to 3PR. Majumder and Groenevelt [18] first analyzed the interaction between 3PR and OEM and discovered that the employment of 3PR has a large impact on the profits of the OEM. Huang and Wang [5] compared different remanufacturing modes and proposed that information sharing can effectively improve the qualities of remanufactured products. Wu and Zhou [6] found that third-party remanufacturing not only increases the income of both the OEM and the remanufacturer but also leads to the improvement of total social welfare. Qiao and Su [7] constructed a two-phase game model to resolve the OEMs’ licensing strategies and the 3PRs’ distribution channel choice issues and found the best royalties for both parties. Zhou and Yuen [19] introduced government subsidies into remanufacturing. Feng et al. [20] studied the trade-in strategy for a CLSC with one manufacturer and two independent remanufacturers. From the above papers, the construction of the production model of authorized remanufacturing not only solves the problems of intellectual property rights and patent licensing but also improves the remanufacturing efficiency and increases the revenue of both the OEMs and 3PRs; consequently, it also leads to the improvement of the overall welfare of society. However, the above-mentioned literature only considers the equilibrium conditions for achieving authorization under different circumstances; it does not consider how the parameters of the model can affect the profits of the decision-makers, the choice of the collection model, and the impact of remanufacturing on environmental pollution. Moreover, there are very few papers considering the model of the OEMs’ in-house remanufacturing, which refers to the situation in which OEMs are responsible for remanufacturing and selling remanufactured products. Therefore, our paper fills the gap in the existing literature by considering two strategies of remanufacturing, namely, the OEMs’ in-house remanufacturing and their authorized remanufacturing. On this basis, the scope of our investigation is expanded to study the strategic choices for the OEM between the two remanufacturing strategies above. We shall analyze the impact of these two remanufacturing strategies on product quantities, prices, profits, and environmental pollution, and shall guide the OEMs on how to improve their production and operation techniques.

### 2.2. Equilibrium Decision-Making in a CLSC Network

The CLSC network is a network structure that comprises multiple OEMs, retailers, and 3PRs [12]. At each level, each OEM (retailer or 3PR) competes non-cooperatively with other OEMs (retailers or 3PRs). The targets for reuse and recycling the used product by OEMs or 3PRs for remanufacturing facilitate reverse flows implemented into the OEMs’ forward product flows, giving rise to the CLSC. Qiang [13] studied a two-period CLSC network and considered the production quantity and the level of manufacturers’ remanufacturability. Zhou et al. [14] studied the remanufacturing policy in a CLSC network based on the recovery rates, remanufacturing costs, and environmental impact. Salehi-Amiri et al. [15] applied the CLSC network to the avocado industry. Chan et al. [21] studied a dynamic equilibrium of the CLSC network to explain the seasonality of demand. Liu et al. [22] studied a dual-channel CLSC network optimization problem considering the net present value of the network, carbon emissions, and social sustainability indicators. Fu et al. [23] used statistics of new energy vehicles in China to study reverse channel problems in dynamic CLSC systems to provide suggestions for the selection of reverse channels. Fu et al. [24] proposed an equilibrium model of a coupled CLSC network, namely the forward and reverse supply chain network, to analyze the interactions between these two networks. The existing literature simply analyzes the equilibrium result of the CLSC network, but it does not distinguish different demands between new products and green products (remanufactured products) in the models, and it does not consider the choice of remanufacturing strategies in the network. Therefore, in this paper, we expand the equilibrium models of the CLSC network by considering the consumers’ green preferences of the new products and the remanufactured products in our model; moreover, the choice of the two strategies of remanufacturing, i.e., in-house remanufacturing and authorized remanufacturing, are also included and separately discuss the equilibrium of the CLSC network considering these two strategies in our model.

### 2.3. Carbon Emission Trading, Green Innovation, and Green Consumers

Besides considering the criteria for the manufacturers to choose between remanufacturing strategies, this paper also analyzes the influence of other factors on the remanufacturing strategies, such as carbon emission trading, green innovation, and the green consumer ratio. In recent years, the economy has developed rapidly, and carbon dioxide emissions have also increased. Thus, many countries continue to call for measures to reduce carbon emissions to curb the global greenhouse effect and transform the world economy into a green and sustainable one. Consequently, Aiying and Risto [25] applied the carbon trading system to large-scale industrial activities; Chai et al. [26] studied how carbon allowances and carbon emissions trading can be conducive to remanufacturing, and discovered that in the long run manufacturers should invest in low carbon production technology; Aldieri et al. [27] found that the green innovation subsidy policy can help the enterprises to adopt to the green technology and increase their level of employment; Huang et al. [28] found that the green credit in a supply chain is good for social welfare and environmental benefit. Lee [29] found that the supply chain members’ participation in green innovation activities could achieve a win-win scenario in the CLSC; Xia et al. [30] found that the emergence of carbon trading systems will affect the low-carbon behavior of the OEMs; Yang et al. [31] studied the selection of remanufacturing collection modes under the carbon emission trading system; Li et al. [32] found that the government subsidy strategy could reduce carbon emissions; Lv and Li [33] studied the influence of green consumers on enterprises’ green innovation and found that enterprises would choose to carry out green innovation when green consumers exist in the market; Wang et al. [34] found that stringent regulations do not imply more remanufacturing; Cheng et al. [35] found that manufacturers will increase their green technology investment level under the carbon emission constraint; Sarkar et al. [36] found that green innovation can increase the sale quantity of the product; Xue et al. [37] found that consumers’ low-carbon awareness could increase the profits of supply chain members; Yang et al. [38] did a series of studies on the introduction of carbon emissions trading into remanufacturing. From the above research, it is evident that under the carbon trading system, producers’ production behaviors are constrained in such a way that they will be actively assuming the social responsibility of reducing carbon emissions. The demand for green products from green consumers can prompt companies to make green innovations. Efforts to carry out green production and green innovation efforts can help manufacturing companies improve their competitiveness. Therefore, this paper studies the influence of these green factors on the choice between the two remanufacturing strategies, and the research on carbon emission trading, green innovation, and green consumers in this paper can provide companies with more realistic decision-making plans, which will help them to actively adopt strategy changes, i.e., in-house remanufacturing and authorized remanufacturing, and contribute to the renewal of the companies by finding a greener production method for remanufacturing. 

### 2.4. Research Methodology

At present, there are few studies on the CLSC network concerning how green factors can affect the choice of the remanufacturing strategies. However, it is evident that carbon trading, green innovation efforts, and green consumers have great impacts on the choice of remanufacturing strategies for manufacturing enterprises. Therefore, in this paper, we introduce green factors to the existing CLSC network models and study the impact of green factors on the choice between two remanufacturing strategies: the in-house remanufacturing strategy and the authorized remanufacturing strategy. Table 1 provides the main characteristics of this paper. We use model analysis to obtain the equilibrium of the CLSC network considering carbon trading, green innovation efforts, and green consumers, whereby OEMs choose either the in-house remanufacturing or the authorized remanufacturing strategy. Additionally, the relationships of these green factors to the decision variables are also discussed. To obtain the equilibrium of the CLSC network, we use a variational inequality (VI) method the same as that used by Nagurney (1999) [39] to analyze the network economics equilibrium. We solve three numerical examples to analyze the impact of carbon trading, green innovation efforts, and green consumers on the choice between the two remanufacturing strategies. Based on the results of this numerical analysis, we provide suggestions to the manufacturing enterprises on how to choose the better remanufacturing strategy under different circumstances.

## 3. Model and Assumption

**Figure 1:**
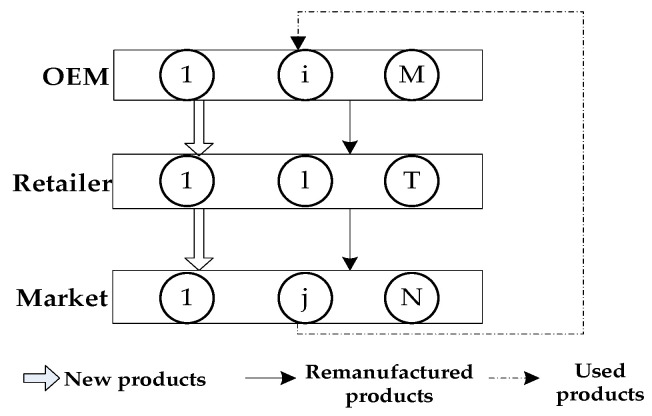
The CLSC network of the IHRS model.

The first model of this paper, entitled the ‘In-House Remanufacturing Strategy (IHRS) model’, corresponds to the situation in which the OEMs choose an IHRS. In this model, the CLSC network consists of M OEMs, T competing retailers, and N demand markets that compete in a Nash [41] non-cooperative manner. The situation is depicted in Figure 1. As shown in Figure 1, the OEMs produce electronic products, such as computers, and they are responsible for the collection and remanufacturing of electronic waste products. Since the preferences of the ordinary and the green customers between purchasing new products and remanufactured (green) products are not the same [42,43], OEMs and retailers sell these two products to ordinary customers only and green customers only, respectively. Moreover, because the government imposes payment to the OEMs for any excess carbon emissions from remanufactured products [42], OEMs need to optimize the use of natural resources by using green innovation efforts. The decision variables, parameters, and cost functions of the IHRS model are shown in Table 2, Table 3 and Table 4, respectively.

**Figure 2:**
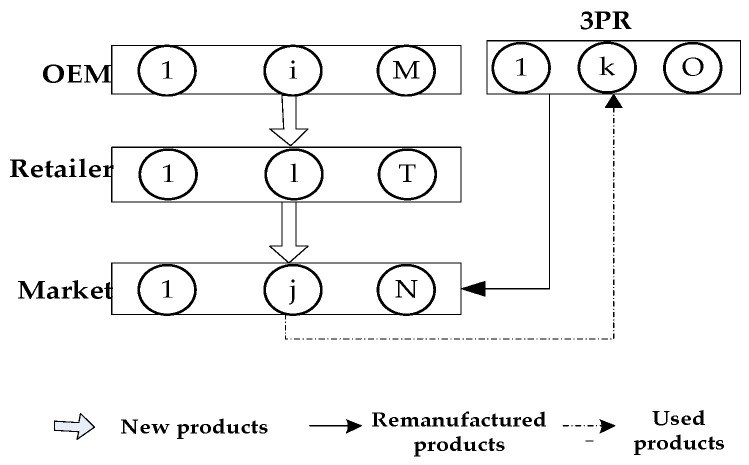
The CLSC network of the ARS model.

The second model of this paper, entitled the ‘Authorized Remanufacturing Strategy (ARS) model’, corresponds to the situation in which OEMs choose the ARS. In this model, the CLSC network consists of M OEMs, T competing retailers, N demand markets, and O 3PRs that compete in a Nash [41] non-cooperative manner. The situation is depicted in Figure 2. Unlike Figure 1, Figure 2 illustrates the situation in which 3PRs are responsible for the collection and remanufacturing of used products such that the remanufactured products are directly sold by them to green consumers with the authorization fees paid to the OEMs. In the same way as the situation in the IHRS model, the new products in this model are sold to ordinary consumers, whereas the remanufactured products are sold to green consumers in the market. Identically to the situation of the IHPR model, since the government imposes payment to both the OEMs and the 3PRs for any excess carbon emissions from remanufactured products [42], both the OEMs and the 3PRs need to optimize the use of natural resources by using green innovation efforts. Since some decision variables and some cost functions in the ARS model have already been defined in the IHRS model, we only define the new decision variables, parameters, and cost functions of the ARS model in Table 5.

We assume throughout this paper that the following conditions are satisfied:

**Assumption 1.**  *Since the preferences of the ordinary and the green customers between purchasing new products and remanufactured (green) products are not the same, the demands for new products and remanufactured products are also different [42,43]. Let the demand for new products and remanufactured products be denoted by $d_{j}^{n}\left(\rho_{j}^{n},\rho_{j}^{r}\right)$ and $d_{j}^{r}\left(\rho_{j}^{n},\rho_{j}^{r}\right)$, respectively, then*

(1) $$d_{j}^{n}=\left(1-\tau \right)\left(1000+100*\chi -\frac{2\rho_{j}^{n}-1.5\rho_{j}^{r}}{1-\delta }\right)$$

(2) $$d_{j}^{r}=\left(1-\tau \right)\left(\frac{1.5\delta \rho_{j}^{n}-2\rho_{j}^{r}}{\delta \left(1-\delta \right)}\right)+\tau \left(1000+100*\chi -10\rho_{j}^{r}\right)$$

*In both (1) and (2), τ represents the proportion of green consumers in the demand market, and χ represents the OEMs’ innovation effort; these two parameters also affect the prices of both the new products and the remanufactured products. Since green consumers are more inclined to buy green products than ordinary consumers, green consumers have a larger impact on the number of remanufactured products produced by the OEMs and the 3PRs than ordinary consumers*.

**Assumption 2.**  *A single production period without considering storage decisions is used. Thus, we assume that only new products can be recycled to become remanufactured products; remanufactured products cannot be further recycled [25]*.

**Assumption 3.**  *The higher the green innovation effects of the OEMs, the larger the number of new products that will be recycled to become remanufactured products [42]*.

**Assumption 4.**  *A carbon trading system for enterprises has been implemented by the government in such a way that the amount of carbon emission from the remanufactured products in a single production period should not exceed an upper limit; when the company’s carbon emission exceeds the upper limit, a penalty cost per unit of excessive carbon emission will be charged to the company; when the company’s carbon emission does not exceed the upper limit, it can sell the available units of carbon emission to other companies to obtain extra profits [38]*.

**Assumption 5.**  *In the ARS model, there should not be too many remanufacturing products in the market, because they will grab the OEMs’ market share [44]. Thus, OEMs and 3PRs have reached an agreement that the number of remanufactured products in a single production should not exceed a certain percentage of the number of used products in the market. (The ratio of the number of remanufactured products in the market to the number of used products in the market is called the remanufacturing ratio.)*.

**Assumption 6.**  *The model in this paper is targeting the electronic product industry only [2]; all the products involved in this model and the numerical examples are all electronic products*.

**Assumption 7.**  *All the cost functions are continuously differentiable convex functions [11]*.

## 4. Model 1: In-House Remanufacturing Strategy

**Figure 3:**
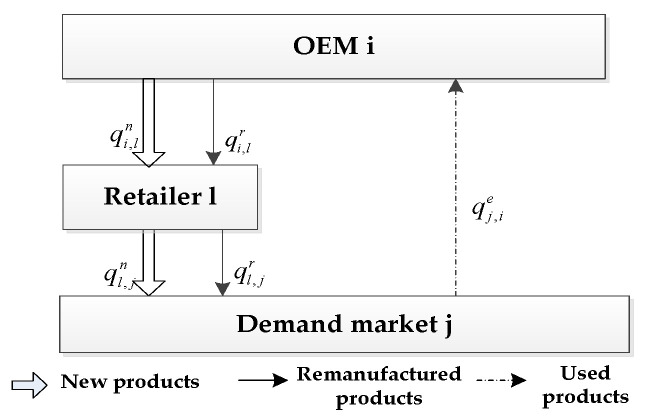
The transaction diagram of the IHRS model.

When the OEM chooses the IHRS as shown in Figure 1, the new product will be produced by the M OEMs and sold to N demand markets through T retailers, whose demand function is given by (1). After the new products have been consumed by the consumers, some of the used products will be recycled by the M OEMs for remanufacturing, and then resold to the N demand market through the T retailers as remanufactured products, which will be sold to green consumers in the markets. The demand function for these remanufactured products is given by (2) and the transaction of each decision-maker is shown in Figure 3.

From Figure 3, during one OEMs’ production period, the transactions between OEM i, retailer l, and demand market j are as follows: OEM i produces $q_{i}^{v}$ items of new products and sells $q_{i,l}^{n}$ items of these new products to retailer l, who will in turn sell $q_{l,j}^{n}$ of them to the demand market j. After the new products have been resumed by the consumers in demand market j, it will return $q_{j,i}^{e}$ items of these used products to the OEM i for remanufacturing. After the used products have been remanufactured by the OEM i, they will become remanufactured products. The OEM i will then sell $q_{i,l}^{r}$ item(s) of these remanufactured products to retailer l, who will in turn sell $q_{l,j}^{r}$ of them to the demand market j. (The consumers can buy the new products or remanufactured products from the demand markets according to their green product preferences.)

### 4.1. Equilibrium Decisions of the OEMs

As mentioned in the previous paragraph, the new products in the network are produced by M OEMs that compete in a non-cooperative manner, which will be sold to N demand markets through T retailers. After the new products have been used by the consumers, they become used products. The used products can be recycled by the OEMs for remanufacturing. After the used products have been remanufactured by the OEMs, they will become green remanufactured products. These green remanufactured products will be sold to the N demand markets via the T retailers to satisfy the demands of the green consumers. For the new products, suppose that in one production period, OEM i produces $q_{i}^{v}$ items of new products with the production cost $f_{i}\left(Q^{v},\chi \right)$, where χ represents the OEMs’ green innovation effort and $\frac{\partial f_{i}\left(Q^{v},\chi \right)}{\partial \chi }>0$, which indicates the higher the OEMs’ green innovation effort, the higher the production cost of new products. Then, OEM i sells $q_{i,l}^{n}$ items of these new products to the retailer l with a transaction cost $c_{i,l}^{n}\left(q_{i,l}^{n}\right)$ and an endogenous price $p_{i,l}^{n}$ for the purchase of new products. After the new products have been consumed by the consumers, OEM i collects $q_{j,i}^{e}$ units of these used products from the demand market j with a transaction cost $c_{j,i}^{e}\left(q_{j,i}^{e}\right)$, a collection cost $\rho_{j}^{e}q_{j,i}^{e},$ and an endogenous price $\rho_{j}^{e}$ for the purchase of used products. Moreover, OEM i also needs to pay the total inspection cost $\phi_{i}\left(\sum_{j=1}^{N}q_{j,i}^{e}\right)$ for the inspection of all used products. After thoroughly inspecting, only $\beta \sum_{j=1}^{N}q_{j,i}^{e}$ items of the used products can be remanufactured. For the remaining $\bar{\beta }\sum_{j=1}^{N}q_{j,i}^{e}$, (where $\bar{\beta }=1-\beta$) items that cannot be remanufactured, they need to be sent to the landfill for disposal. Thus, the OEM i also needs to pay the total transportation cost $c_{i,D}\left(\bar{\beta }\sum_{j=1}^{N}q_{j,i}^{e}\right)$ for transferring the waste products from all the demand markets to the landfill site, and the landfill cost $\bar{\beta }\bar{\rho }\sum_{j=1}^{N}q_{j,i}^{e}$ for the disposal of all the waste products at the landfill. For the remanufactured products, suppose that in one production period, the OEM i sells $q_{i,l}^{r}$ items of the remanufactured products to the retailer l with a transaction cost $c_{i,l}^{r}\left(q_{i,l}^{r}\right)$ and an endogenous price $p_{i,l}^{r}$. Moreover, the OEM i also needs to pay the total production cost $r_{i}\left(\sum_{l=1}^{T}q_{i,l}^{r},\chi \right)$ for the remanufacturing of all the used products, where $\frac{\partial r_{i}\left(\sum_{l=1}^{T}q_{i,l}^{r},\chi \right)}{\partial \chi }<0$, which indicates the higher the OEMs’ green innovation effort, the lower the production cost of the remanufactured products. As mentioned in Assumption 4, a carbon emission and trading system for enterprises has been implemented by the government in such a way that the amount of carbon emission from the remanufactured products in a single production should not exceed an upper limit; thus, when the company’s carbon emissions from the production of new or remanufactured products exceeds the upper limit $\bar{M}$, it needs to pay, respectively, the carbon emission costs of $\nu \left(e_{n}q^{v}-\bar{M}\right)$ for the new products and $\nu \left(e_{r}\sum_{l=1}^{T}q_{i,l}^{r}-\bar{M}\right)$ for the remanufactured products, where $e_{n}$ and $e_{r}$ are the environmental impact of one item of the new products and the remanufactured product, respectively. $e_{n}q_{i}^{v}$ and $e_{r}\sum_{l=1}^{T}q_{i,l}^{r}$ are, respectively, the amount of the company’s carbon emission for these two products in one production cycle. This carbon emission and trading system for enterprises is consistent with that given in the life cycle assessment (LCA) [45]. 

Thus, the income of the OEM i includes that obtained by selling these two products to the T retailers, whereas his/her costs include the production cost, the recycling cost, the transaction cost, and the carbon emission cost. Thus, the profit of OEM i, denoted by $\Pi_{i}^{1}$, can be expressed as follows:

(3) $$\begin{array}{l} \max\Pi_{i}^{1}=\sum_{l=1}^{T}p_{i,l}^{n}q_{i,l}^{n}-f_{i}\left(Q^{v},\chi \right)-\sum_{l=1}^{T}c_{i,l}^{n}\left(q_{i,l}^{n}\right)-\sum_{j=1}^{N}\rho_{j}^{e}q_{j,i}^{e}-\sum_{j=1}^{N}c_{j,i}^{e}\left(q_{j,i}^{e}\right)\\ -\phi_{i}\left(\sum_{j=1}^{N}q_{j,i}^{e}\right)-c_{i,D}\left(\bar{\beta }\sum_{j=1}^{N}q_{j,i}^{e}\right)-\bar{\beta }\bar{\rho }\sum_{j=1}^{N}q_{j,i}^{e}+\sum_{l=1}^{T}p_{i,l}^{r}q_{i,l}^{r}-r_{i}\left(\sum_{l=1}^{T}q_{i,l}^{r},\chi \right)-\\ \sum_{l=1}^{T}c_{i,l}^{r}\left(q_{i,l}^{r}\right)-v\left(e_{n}q_{i}^{v}-\bar{M}\right)-v\left(e_{r}\sum_{l=1}^{T}q_{i,l}^{r}-\bar{M}\right) \end{array}$$

(4) $$s.t.\;\sum_{l=1}^{T}q_{i,l}^{n}\leq q_{i}^{v}$$

(5) $$\alpha \sum_{l=1}^{T}q_{i,l}^{n}\leq \sum_{j=1}^{N}q_{j,i}^{e}$$

(6) $$\sum_{l=1}^{T}q_{i,l}^{r}\leq \beta \sum_{j=1}^{N}q_{j,i}^{e}$$

(7) $$q_{i}^{v},q_{i,l}^{n},q_{i,l}^{r},q_{j,i}^{e}\geq 0,\forall l,\forall j$$

Constraint (4) indicates that the sales of all new products cannot exceed their production quantity; constraint (5) means that the total recycled products cannot be less than the minimum specified quantity of new products, where α is the lower limit of the recovery rate; constraint (6) indicates that the total remanufactured products cannot exceed the total amount of used amount that can be recovered from all the markets for recycling, where β is the fraction of used products that can be used for remanufacturing.

**Proposition 1.**  *In the IHS model, the profit function of OEM i is concave*.

**Proof.**  See Appendix A. □

**Theorem 1.**  Assume that in the IHRS model, all the OEMs are competing in a non-cooperative manner. Then from Proposition 1 and Assumption 7, the equilibrium of all the OEMs satisfies the VI [39] as defined in (8). In other words, find $\left(Q^{v*},Q^{1*},Q^{2*},Q^{5*},\mu^{*},\varepsilon^{*},\varphi^{*}\right)\in R_{+}^{M+2MT+NM+3M}$ (The equilibrium decisions are denoted by “*”), which satisfies the following VI:

(8) $$\begin{array}{l} \sum_{i=1}^{M}\left[\frac{\partial f_{i}\left(Q^{v*},\chi \right)}{\partial q_{i}^{v}}-\varepsilon_{i}^{*}+ve_{n}\right]\times \left[q_{i}^{v}-q_{i}^{v*}\right]+\sum_{i=1}^{M}\sum_{l=1}^{T}\left[-p_{i,l}^{n*}+\frac{\partial c_{i,l}^{n}\left(q_{i,l}^{n*}\right)}{\partial q_{i,l}^{n}}+\alpha \mu_{i}^{*}+\varepsilon_{i}^{*}\right]\times \left[q_{i,l}^{n}-q_{i,l}^{n*}\right]\\ +\sum_{i=1}^{M}\sum_{l=1}^{T}\left[-p_{i,l}^{r*}+\frac{\partial r_{i}\left(\sum_{l=1}^{T}q_{i,l}^{r*},\chi \right)}{\partial q_{i,l}^{r}}+\frac{\partial c_{{}_{i,l}}^{r}\left(q_{i,l}^{r*}\right)}{\partial q_{i,l}^{r}}+\varphi_{i}^{*}+ve_{r}\right]\times \left[q_{i,l}^{r}-q_{i,l}^{r*}\right]\\ +\sum_{j=1}^{N}\sum_{i=1}^{M}\left[\rho_{j}^{e*}+\frac{\partial c_{j,i}^{e}\left(q_{j,i}^{e*}\right)}{\partial q_{j,i}^{e}}+\frac{\partial \phi_{i}\left(\sum_{j=1}^{N}q_{j,i}^{e*}\right)}{\partial q_{j,i}^{e}}+\frac{\partial c_{i,D}\left(\bar{\beta }\sum_{j=1}^{N}q_{j,i}^{e*}\right)}{\partial q_{j,i}^{e}}+\bar{\beta }\bar{\rho }-\mu_{i}^{*}-\beta \varphi_{i}^{*}\right]\times \left[q_{j,i}^{e}-q_{j,i}^{e*}\right]\\ +\sum_{i=1}^{M}\left[q_{i}^{v*}-\sum_{l=1}^{t}q_{i,l}^{n*}\right]\times \left[\varepsilon_{i}-\varepsilon_{i}^{*}\right]+\sum_{i=1}^{M}\left[\sum_{j=1}^{N}q_{j,i}^{e*}-\alpha \sum_{l=1}^{T}q_{i,l}^{n*}\right]\times \left[\mu_{i}-\mu_{i}^{*}\right]+\sum_{i=1}^{M}\left[\beta \sum_{j=1}^{N}q_{j,i}^{e*}-\sum_{l=1}^{T}q_{i,l}^{r*}\right]\times \left[\varphi_{i}-\varphi_{i}^{*}\right]\geq 0,\\ \forall \left(Q^{v},Q^{1},Q^{2},Q^{5},\varepsilon ,\mu ,\varphi \right)\in R_{+}^{M+2MT+NM+3M}. \end{array}$$

*In VI (8), $\varepsilon_{i}$, $\mu_{i}$ and $\varphi_{i}$ are the Lagrange multipliers of (4), (5), and (6), respectively*.

**Proof.**  See Appendix B. □

### 4.2. Equilibrium Decisions of the Retailers

As mentioned earlier, T retailers competing non-cooperatively in the network purchase new products and remanufactured products from M OEMs and sell them to N demand markets to satisfy the demands of the consumers. Suppose that during one OEM’s production period, retailer l purchases $q_{i,l}^{n}$ items of new products and $q_{i,l}^{r}$ items of remanufactured products from the OEM i with the endogenous price of $p_{i,l}^{n}$ and $p_{i,l}^{r}$, respectively. Moreover, the retailer l also needs to pay the exhibition costs $c_{l}^{n}\left(Q^{1}\right)$ and $c_{l}^{r}\left(Q^{2}\right)$ for the sale of these two products, respectively. The retailer l then sells $q_{l,j}^{n}$ items of the new products and $q_{l,j}^{r}$ items of the remanufactured products to the demand market j with the endogenous prices of $p_{l,j}^{n}$ and $p_{l,j}^{r}$, respectively. Thus, the income of the retailer l includes those obtained by selling these two products to the N demand markets, whereas his/her costs include the purchasing costs and the exhibition costs of these two products. Thus, the net profit of the retailer l, denoted by $\Pi_{l}^{1}$, can be expressed as follows:

(9) $$\max\Pi_{l}^{1}=\sum_{j=1}^{N}p_{l,j}^{n}q_{l,j}^{n}+\sum_{j=1}^{N}p_{l,j}^{r}q_{l,j}^{r}-\sum_{i=1}^{M}p_{i,l}^{n}q_{i,l}^{n}-\sum_{i=1}^{M}p_{i,l}^{r}q_{i,l}^{r}-c_{l}^{n}\left(Q^{1}\right)-c_{l}^{r}\left(Q^{2}\right)$$

(10) $$s.t.\sum_{j=1}^{N}q_{l,j}^{n}\leq \sum_{i=1}^{M}q_{i,l}^{n}$$

(11) $$\sum_{j=1}^{N}q_{l,j}^{r}\leq \sum_{i=1}^{M}q_{i,l}^{r}$$

(12) $$q_{i,l}^{n},q_{i,l}^{r},q_{l,j}^{n},q_{l,j}^{r}\geq 0,\;\forall j,\forall i$$

Constraints (10) and (11) indicate that the total amount of these two products sold by retailers to all the markets cannot exceed the total amount purchased from all the OEMs.

**Proposition 2.**  *In the IHRS model, the profit function of retailer l is a concave function of its decision variables*.

**Proof.**  The proof of this theorem can be obtained from that of Proposition 1. □

**Theorem 2.**  Assume that in the IHRS model, all the retailers are competing in a non-cooperative manner. Then, from Proposition 2 and Assumption 7, the equilibrium of all the retailers satisfies the VI as defined in (13). In other words, find $\left(Q^{1*},Q^{2*},Q^{3*},Q^{4*},\gamma^{*},\eta^{*}\right)\in R_{+}^{2MT+2TN+2T}$ which satisfies the following VI:

(13) $$\begin{array}{l} \sum_{i=1}^{M}\sum_{l=1}^{T}\left[p_{i,l}^{n*}+\frac{\partial c_{l}^{n}\left(Q^{1*}\right)}{\partial q_{i,l}^{n}}-\gamma_{l}^{*}\right]\times \left[q_{i,l}^{n}-q_{i,l}^{n*}\right]+\sum_{i=1}^{M}\sum_{l=1}^{T}\left[p_{i,l}^{r*}+\frac{\partial c_{l}^{r}\left(Q^{2*}\right)}{\partial q_{i,l}^{r}}-\eta_{l}^{*}\right]\times \\ \left[q_{i,l}^{r}-q_{i,l}^{r*}\right]+\sum_{l=1}^{T}\sum_{j=1}^{N}\left[-p_{l,j}^{n*}+\gamma_{l}^{*}\right]\times \left[q_{l,j}^{n}-q_{l,j}^{n*}\right]+\sum_{l=1}^{T}\sum_{j=1}^{N}\left[-p_{l,j}^{r*}+\eta_{l}^{*}\right]\times \left[q_{l,j}^{r}-q_{l,j}^{r*}\right]+\\ \sum_{l=1}^{T}\left[\sum_{i=1}^{M}q_{i,l}^{n*}-\sum_{j=1}^{N}q_{l,j}^{n*}\right]\times \left[\gamma_{l}-\gamma_{l}^{*}\right]+\sum_{l=1}^{T}\left[\sum_{i=1}^{M}q_{i,l}^{r*}-\sum_{j=1}^{N}q_{l,j}^{r*}\right]\times \left[\eta_{l}-\eta_{l}^{*}\right]\geq 0,\\ \forall \left(Q^{1},Q^{2},Q^{3},Q^{4},\gamma ,\eta \right)\in R_{+}^{2MT+2TN+2T} \end{array}$$

*In VI (13), $\gamma_{l}$ and $\eta_{l}$ are the multipliers of (10) and (11), respectively*.

**Proof.**  The proof of this theorem is similar to of Theorem 1. □

### 4.3. The Equilibrium Decisions of the Demand Markets

When the OEMs choose the IHRS, the consumers in each of the N demand markets can be classified as ordinary consumers and green consumers, who choose to purchase new products and remanufacturing products from retailers, respectively.

According to [12], for the transaction of new products in the demand market j, the price of the new products $\rho_{j}^{n}$ and the sales of new products $q_{l,j}^{n}$ should satisfy the following complementary problems (CPs):

(14) $$p_{l,j}^{n}+c_{l,j}^{n}\left(q_{l,j}^{n}\right)\left\{ \begin{array}{l} =\rho_{j}^{n},q_{l,j}^{n}>0\\ \geq \rho_{j}^{n},q_{l,j}^{n}=0 \end{array}\right.$$

(15) $$d_{j}^{n}\left(\rho_{j}^{n},\rho_{j}^{r}\right)\left\{ \begin{array}{l} =\sum_{l=1}^{T}q_{l,j}^{n}\;\;if\;\rho_{j}^{n}>0\\ \leq \sum_{l=1}^{T}q_{l,j}^{n}\;\;if\;\rho_{j}^{n}=0 \end{array}\right.$$

In (14), the demand for new products $d_{j}^{n}\left(\rho_{j}^{n},\rho_{j}^{r}\right)$ should satisfy (1). CP (14) indicates that if the consumers from the demand market j are willing to purchase new products from the retailer l, then the price they need to pay for these new products, $\rho_{j}^{n}$, is equal to the sum of the purchase cost and the transaction cost of these new products between the retailer l and the demand market j. CP (15) indicates that if the consumers from the demand market j are willing to purchase new products, then the demand for new products is equal to the total new products purchased by the demand market j from all the retailers.

Since only new products can be recycled for further use and remanufactured products cannot be further recycled, the activities of the reverse logistics in the CLSC network apply to new products only. Thus, after the new products have been consumed by the consumers and become used products, they are recycled and remanufactured by the M OEMs. Hence, if the consumers from the demand market j are willing to return the used products, then the used products returned to the OEM i from the demand market $j,\;\left(i.e.,\;\sum_{i=1}^{M}q_{j,i}^{e}\right)$ should satisfy the following CP:

(16) $$a_{j}\left(\sum_{i=1}^{M}q_{j,i}^{e}\right)\left\{ \begin{array}{l} =\rho_{j}^{e}\;if\;q_{j,i}^{e}>0\\ \geq \rho_{j}^{e}\;if\;q_{j,i}^{e}=0 \end{array}\right.\;$$

(17) $$s.t.\sum_{i=1}^{M}q_{j,i}^{e}\leq \sum_{l=1}^{T}q_{l.j}^{n}$$

(18) $$q_{l,j}^{n},q_{j,i}^{e}\geq 0,\;\forall i,\forall l$$

Constraint (17) indicates that the used products returned from the demand market j to all the OEMs should not exceed the total new products sold by all the retailers to the demand market j.

Like the transaction of new products in the demand market j, the price of the remanufactured products $\rho_{j}^{r}$ and the demand for remanufactured products $d_{j}^{r}\left(\rho_{j}^{n},\rho_{j}^{r}\right)$ should satisfy the following complementary problems (CPs):

(19) $$p_{l,j}^{r}+c_{l,j}^{r}\left(q_{l,j}^{r}\right)\left\{ \begin{array}{l} =\rho_{j}^{r},q_{l,j}^{r}>0\\ \geq \rho_{j}^{r},q_{l,j}^{r}=0 \end{array}\right.$$

(20) $$d_{j}^{r}\left(\rho_{j}^{n},\rho_{j}^{r}\right)\left\{ \begin{array}{l} =\sum_{l=1}^{T}q_{l,j}^{r}\;\;if\;\rho_{j}^{r}>0\\ \leq \sum_{l=1}^{T}q_{l,j}^{r}\;\;if\;\rho_{j}^{r}=0 \end{array}\right.$$

In (20), the demand for new products $d_{j}^{r}\left(\rho_{j}^{n},\rho_{j}^{r}\right)$ should satisfy (2).

The equilibrium of all the demand markets can be obtained by using the equivalence between CP and VI given by [46].

**Theorem 3.**  In the IHRS model, the equilibrium of all the demand markets is obtained by solving the following VI: Find $\left(Q^{3*},Q^{4*},Q^{5*},\rho^{n*},\rho^{r*},\lambda^{*}\right)\in R_{+}^{2TN+NM+3N}$ which satisfies:

(21) $$\begin{array}{l} \sum_{l=1}^{T}\sum_{j=1}^{N}\left[p_{l,j}^{n*}+c_{l,j}^{n}\left(q_{l,j}^{n*}\right)-\rho_{j}^{n*}-\lambda_{j}^{*}\right]\times \left[q_{l,j}^{n}-q_{l,j}^{n*}\right]+\sum_{l=1}^{T}\sum_{j=1}^{N}\left[p_{l,j}^{r*}+c_{l,j}^{r}\left(q_{l,j}^{r*}\right)-\rho_{j}^{r*}\right]\times \left[q_{l,j}^{r*}-q_{l,j}^{r*}\right]\\ +\sum_{j=1}^{N}\sum_{i=1}^{M}\left[a_{j}\left(\sum_{i=1}^{M}q_{j,i}^{e*}\right)-\rho_{j}^{e*}+\lambda_{j}^{*}\right]\times \left[q_{j,i}^{e}-q_{j,i}^{e*}\right]+\sum_{j=1}^{N}\left[\sum_{l=1}^{T}q_{l,j}^{n*}-d_{j}^{n}\left(\rho_{j}^{n*},\rho_{j}^{r*}\right)\right]\times \left[\rho_{j}^{n}-\rho_{j}^{n*}\right]\\ +\sum_{j=1}^{N}\left[\sum_{l=1}^{T}q_{l,j}^{r*}-d_{j}^{r}\left(\rho_{j}^{n*},\rho_{j}^{r*}\right)\right]\times \left[\rho_{j}^{r}-\rho_{j}^{r*}\right]+\sum_{j=1}^{N}\left[\sum_{l=1}^{T}q_{l.j}^{n*}-\sum_{k=1}^{O}q_{j,i}^{e*}\right]\times \left[\lambda_{j}-\lambda_{j}^{*}\right]\geq 0,\\ \forall \left(Q^{3},Q^{4},Q^{5},\rho^{n},\rho^{r},\lambda \right)\in R_{+}^{2NT+NM+3N}. \end{array}$$

*In VI (21), $\lambda_{j}$ is the multiplier of (17)*.

### 4.4. Equilibrium of the Network

Suppose the decisions of all the decision-makers are at equilibrium simultaneously, then VI (8), (13), and (21) hold simultaneously. By adding these inequalities together and subtracting all the endogenous prices ($p_{i,l}^{n}$, $p_{i,l}^{r}$, $p_{l,j}^{n}$, $p_{l,j}^{r}$, and $\rho_{j}^{e}$), the equilibrium of the network is as follows:

**Theorem 4.**  In the IHRS model, the equilibrium of the network is obtained from the following VI: Find $\left(Q^{v*},Q^{1*},Q^{2*},Q^{3*},Q^{4*},Q^{5*},\rho^{n*},\rho^{r*},\mu^{*},\varepsilon^{*},\varphi^{*},\gamma^{*},\eta^{*},\lambda^{*}\right)\in R_{+}^{M+2MT+2TN+NM+2N+3M+2T+N}$, which satisfies:

(22) $$\begin{array}{l} \sum_{i=1}^{M}\left[\frac{\partial f_{i}\left(Q^{v*},\chi \right)}{\partial q_{i}^{v}}-\varepsilon_{i}^{*}+ve_{n}\right]\times \left[q_{i}^{v}-q_{i}^{v*}\right]+\sum_{i=1}^{M}\sum_{l=1}^{T}\left[\frac{\partial c_{i,l}^{n}\left(q_{i,l}^{n*}\right)}{\partial q_{i,l}^{n}}+\alpha \mu_{i}^{*}+\varepsilon_{i}^{*}+\frac{\partial c_{l}^{n}\left(Q^{1*}\right)}{\partial q_{i,l}^{n}}-\gamma_{l}^{*}\right]\times \left[q_{i,l}^{n}-q_{i,l}^{n*}\right]\\ +\sum_{i=1}^{M}\sum_{l=1}^{T}\left[\frac{\partial r_{i}\left(\sum_{l=1}^{T}q_{i,l}^{r*},\chi \right)}{\partial q_{i,l}^{r}}+\frac{\partial c_{i,l}^{r}\left(q_{i,l}^{r*}\right)}{\partial q_{i,l}^{r}}+\varphi_{i}^{*}+ve_{r}+\frac{\partial c_{l}^{r}\left(Q^{2*}\right)}{\partial q_{i,l}^{r}}-\eta_{l}^{*}\right]\times \left[q_{i,l}^{r}-q_{i,l}^{r*}\right]+\\ \sum_{l=1}^{T}\sum_{j=1}^{N}\left[\gamma_{l}^{*}+c_{l,j}^{n}\left(q_{l,j}^{n*}\right)-\rho_{j}^{n*}-\lambda_{j}^{*}\right]\times \left[q_{l,j}^{n}-q_{l,j}^{n*}\right]+\sum_{l=1}^{T}\sum_{j=1}^{N}\left[\eta_{l}^{*}+c_{l,j}^{r}\left(q_{l,j}^{r*}\right)-\rho_{j}^{r*}\right]\times \left[q_{l,j}^{r}-q_{l,j}^{r*}\right]\\ +\sum_{j=1}^{N}\sum_{i=1}^{M}\left[\frac{\partial c_{j,i}^{e}\left(q_{j,i}^{e*}\right)}{\partial q_{j,i}^{e}}+\frac{\partial \phi_{i}\left(\sum_{j=1}^{N}q_{j,i}^{e*}\right)}{\partial q_{j,i}^{e}}+\frac{\partial c_{i,D}\left(\bar{\beta }\sum_{j=1}^{N}q_{j,i}^{e*}\right)}{\partial q_{j,i}^{e}}+\bar{\beta }\bar{\rho }-\mu_{i}^{*}-\beta \varphi_{i}^{*}+a_{j}\left(\sum_{i=1}^{M}q_{j,i}^{e*}\right)+\lambda_{j}^{*}\right]\times \\ \left[q_{j,i}^{e}-q_{j,i}^{e*}\right]+\sum_{j=1}^{N}\left[\sum_{l=1}^{T}q_{l,j}^{n*}-d_{j}^{n}\left(\rho_{j}^{n*},\rho_{j}^{r*}\right)\right]\times \left[\rho_{j}^{n}-\rho_{j}^{n*}\right]+\sum_{j=1}^{N}\left[\sum_{l=1}^{T}q_{l,j}^{r*}-d_{j}^{r}\left(\rho_{j}^{n*},\rho_{j}^{r*}\right)\right]\times \\ \left[\rho_{j}^{r}-\rho_{j}^{r*}\right]+\sum_{i=1}^{M}\left[\sum_{j=1}^{N}q_{j,i}^{e*}-\alpha \sum_{l=1}^{t}q_{i,l}^{n*}\right]\times \left[\mu_{i}-\mu_{i}^{*}\right]+\sum_{i=1}^{M}\left[q_{i}^{v*}-\sum_{l=1}^{t}q_{i,l}^{n*}\right]\times \left[\varepsilon_{i}-\varepsilon_{i}^{*}\right]+\\ \sum_{i=1}^{M}\left[\beta \sum_{j=1}^{N}q_{j,i}^{e*}-\sum_{l=1}^{T}q_{i,l}^{r*}\right]\times \left[\varphi_{i}-\varphi_{i}^{*}\right]+\sum_{l=1}^{T}\left[\sum_{i=1}^{M}q_{i,l}^{n*}-\sum_{j=1}^{N}q_{l,j}^{n*}\right]\times \left[\gamma_{l}-\gamma_{l}^{*}\right]+\\ \sum_{l=1}^{T}\left[\sum_{i=1}^{M}q_{i,l}^{r*}-\sum_{j=1}^{N}q_{l,j}^{r*}\right]\times \left[\eta_{l}-\eta_{l}^{*}\right]+\sum_{j=1}^{N}\left[\sum_{l=1}^{T}q_{l.j}^{n*}-\sum_{i=1}^{M}q_{j,i}^{e*}\right]\times \left[\lambda_{j}-\lambda_{j}^{*}\right]\geq 0\\ \forall \left(Q^{v},Q^{1},Q^{2},Q^{3},Q^{4},Q^{5},\rho^{n},\rho^{r},\mu ,\varepsilon ,\varphi ,\gamma ,\eta ,\lambda \right)\in R_{+}^{M+2MT+2TN+NM+2N+3M+2T+N}. \end{array}$$

The existence and uniqueness of VI (22) are obtained from the fact that the functions in VI (22) are monotone and Lipschitz continuous [46].

From the equivalence of VI (8) and CP [46], we obtain the prices of these two products sold by OEM i to retailer l at equilibrium as follows:

(23) $$p_{i,l}^{n*}=\frac{\partial c_{i,l}^{n}\left(q_{i,l}^{n*}\right)}{\partial q_{i,l}^{n}}+\alpha \mu_{i}^{*}+\varepsilon_{i}^{*}$$

(24) $$p_{i,l}^{r*}=\frac{\partial r_{i}\left(\sum_{l=1}^{T}q_{i,l}^{r*},\chi \right)}{\partial q_{i,l}^{r}}+\frac{\partial c_{i,l}^{r}\left(q_{i,l}^{r*}\right)}{\partial q_{i,l}^{r}}+\varphi_{i}^{*}+ve_{r}$$

Similarly, we obtain the other endogenous prices at equilibrium as follows:

(25) $$p_{l,j}^{n*}=\gamma_{l}^{*}=\rho_{j}^{n*}-\lambda_{j}^{*}-c_{l,j}^{n}\left(q_{l,j}^{n*}\right)$$

(26) $$p_{l,j}^{r*}=\eta_{l}^{*}=\rho_{j}^{r*}-c_{l,j}^{r}\left(q_{l,j}^{r*}\right)$$

(27) $$\rho_{j}^{e*}=a_{j}\left(\sum_{i=1}^{M}q_{j,i}^{e*}\right)-\lambda_{j}^{*}$$

**Proposition 3.**  *In the IHRS model, the decision variables, i.e., $q_{i}^{v}$, $q_{j,i}^{e}$, $q_{i,l}^{n}$, and $q_{i,l}^{r}$, are decreasing functions of the parameter ν, where ν is the unit price of carbon trading fixed by the government*.

**Proof.**  See Appendix C. □

**The Economic Explanation of Proposition 3.**  Under the influence of the carbon trading system, OEMs are forced to reduce the production of new products to decrease the cost of carbon emissions, resulting in a reduction in the number of new products flowing into the CLSC network and a reduction in the amount of recycling; thus, the number of remanufactured products in the CLSC network also decreases.

**Proposition 4.**  *In the IHRS model, the decision variables, i.e., $q_{i}^{v}$, $q_{j,i}^{e}$, $q_{i,l}^{n}$, and $q_{i,l}^{r}$, are increasing functions of the parameter χ, where χ is the OEMs’ green innovation efforts*.

**Proof.**  The proof can be obtained from that of Proposition 3. □

**The Economic Explanation of Proposition 4.**  The green innovation efforts of the OEMs strive to bring companies closer to consumers in terms of product maintenance and services. Thus, green innovation efforts of the OEMs develop a good reputation for the OEMs, which in term increases consumers’ willingness to buy companies’ products and reduces the remanufacturing cost of the OEMs. Consequently, consumers’ demand for products increases, forming a virtuous circle in the network.

**Proposition 5.**  *In the IHRS model, the decision variables, i.e., $q_{i}^{v}$, $q_{j,i}^{e}$, $q_{i,l}^{n}$, and $q_{i,l}^{r}$, are decreasing functions of the parameter τ, where τ is the proportion of the green consumers in the markets*.

**Proof.**  The proof is similar to that of Proposition 1. □

**The Economic Explanation of Proposition 5.**  The existence of green consumers in the demand markets will affect the production decisions of the OEMs. Thus, the consumers’ demand for green products will cause OEMs to pay attention to the production of remanufactured products. However, enterprises may produce substandard remanufactured products, and some consumers will refuse to buy them. As a result, the demand for these two products will be decreased.

## 5. Model 2: Authorized Remanufacturing Strategy

**Figure 4:**
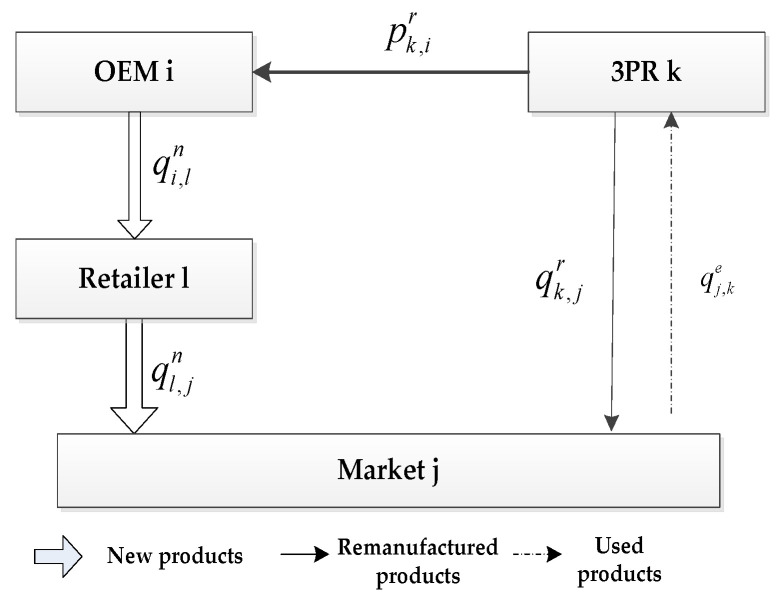
The transaction diagram of the ARS model.

When the OEM chooses the authorized remanufacturing strategy (ARS) as shown in Figure 2, the activities in the forward logistics of this model are the same as those described in the IHRS model. In other words, in the forward logistics, the new products are still produced by M OEMs and sold to N demand markets, whose demand function is given by Equation (1). The differences in the activities between the ARS model and the IHRS model in the reverse logistics are as follows: after the new products have been consumed by the consumers in the ARS model, some of the used products will be recycled by O competing 3PRs, instead of by M competing OEMs, for remanufacturing. The remanufactured products in the ARS model will be sold directly to the demand markets, instead of sold to the demand markets via the retailers, to satisfy the demands of the green consumers. The demand function for the green remanufactured products of the ARS model is given in (2) and the transaction of each decision-maker is shown in Figure 4.

From Figure 3 and Figure 4, identical to the IHRS model, the OEM i in the ARS model also produces $q_{i}^{v}$ items of new products in one production cycle and then sells $q_{i,l}^{n}$ items of these new products to the retailer l. However, unlike the IHRS model, after the new products have been consumed by the consumers, the used products in the ARS model are collected by the 3PRs, instead of by the OEMs, for remanufacturing. Thus, in the ARS model, 3PR k will collect $q_{j,k}^{e}$ items of the used products from the demand market j in one production cycle for remanufacturing. After they have been remanufactured and become green remanufactured products, $q_{k,j}^{r}$ items of these green remanufactured products will be directly sold to the demand market j (The consumers can buy the new products or remanufactured products according to their green product preferences).

### 5.1. Equilibrium of the OEMs

As mentioned in the previous paragraph, new products in the network of the ARS model are produced by M OEMs, which will be sold to N demand markets through T retailers. After the new products have been used by the consumers, they become used products. The OEMs will authorize the 3PRs to collect and remanufacture these used products from the demand markets for which an endogenous patent licensing fee of $p_{k,i}^{s}$ per item will be charged by the OEM i to the 3PR k for remanufacturing. The remanufactured products in the ARS model will be sold directly to the demand markets, instead of being sold to the demand markets via the retailers, to satisfy the demands of the green consumers. Suppose that the OEM i in the ARS model also produces $q_{i}^{v}$ items of new products in one production cycle with production cost $f_{i}\left(Q^{v},\chi \right)$, where χ represents the OEMs’ green innovation effort and $\frac{\partial f_{i}\left(Q^{v},\chi \right)}{\partial \chi }>0$, which indicates the higher the OEMs’ green innovation effort, the higher the production cost of the new products. Then, the OEM i in the ARS model also sells $q_{i,l}^{n}$ items of these new products to the retailer l with a transaction cost $c_{i,l}^{n}\left(q_{i,l}^{n}\right)$ and an endogenous price $p_{i,l}^{n}$ for the purchase of new products.

Moreover, in the same as way as the discussion in the IHRS model, when the company’s carbon emission of new or remanufactured products in the ARS model exceeds the upper limit $\bar{M}$, it also needs to pay, respectively, the carbon emission costs of $\nu \left(e_{n}q_{i}^{v}-\bar{M}\right)$ to produce new products, where $e_{n}$ is the environmental impact of one item of the new products and $e_{n}q_{i}^{v}$ is the amount of the company’s carbon emission from the production of the new products in one production cycle. This carbon emission and trading system for enterprises is consistent with that given in the life cycle assessment (LCA) [45].

When OEM i chooses the ARS, his/her income includes that obtained by selling new products to the T retailers, together the patent licensing fees obtained from the O 3PRs, whereas his/her costs include the carbon emission cost under the carbon trading system. Thus, the net profit of the OEM i in this model, denoted by $\prod_{i}^{2}$, can be expressed as follows:

(28) $$\max\Pi_{i}^{2}=\sum_{l=1}^{T}p_{i,l}^{n}q_{i,l}^{n}-f_{i}\left(Q^{v},\chi \right)-\sum_{l=1}^{T}c_{i,l}^{n}\left(q_{i,l}^{n}\right)+\sum_{k=1}^{O}p_{k,i}^{s}q_{k,i}^{r}-v\left(e_{n}q_{i}^{v}-\bar{M}\right)$$

(29) $$s.t.\sum_{l=1}^{T}q_{i,l}^{n}\leq q_{i}^{v}$$

(30) $$\sum_{k=1}^{O}q_{k,i}^{r}\leq \vartheta \cdot \beta \sum_{l=1}^{T}q_{i,l}^{n}$$

(31) $$q_{i}^{v},q_{i,l}^{n},q_{k,i}^{r}\geq 0,\;\forall l,\forall k$$

Constraint (29) indicates that the sale of new products cannot exceed its production quantity. According to Assumption 5, Constraint (30) indicates that the sale of used products manufactured by all the O 3PRs cannot exceed the maximum remanufacturing quantity specified by the OEM i, i.e., only a proportion ϑ of the total quantity of recoverable new products from OEM i$\left(\beta \sum_{l=1}^{T}q_{i,l}^{n}\right)$ is allowed to be remanufactured, where ϑ is the remanufacturing ratio fixed by the OEMs to the 3PRs, and β is the fraction of used products that can be used for remanufacturing.

**Proposition 6.**  *In the ARS model, the profit function of OEM i is a concave function of its decision variables*.

**Proof.**  The proof is similar to that of Proposition 1. □

**Theorem 5.**  Assume that in the ARS model, all the OEMs are competing in a non-cooperative manner. Then, from Proposition 6 and Assumption 7, the equilibrium of all OEMs satisfies the VI as defined in (32). In other words, find $\left(Q^{v*},Q^{1*},Q^{7*},\sigma^{*},\varpi^{*}\right)\in R_{+}^{M+MT+OM+2M}$ which satisfies the following VI:

(32) $$\begin{array}{l} \sum_{i=1}^{M}\left[\frac{\partial f_{i}\left(Q^{v*},\chi \right)}{\partial q_{i}^{v}}-\sigma_{i}^{*}+ve_{n}\right]\times \left[q_{i}^{v}-q_{i}^{v*}\right]+\sum_{i=1}^{M}\sum_{l=1}^{T}\left[-p_{i,l}^{n*}+\frac{\partial c_{i,l}^{n}\left(q_{i,l}^{n*}\right)}{\partial q_{i,l}^{n}}+\sigma_{i}^{*}-\beta \varpi_{i}^{*}\right]\times \\ \left[q_{i,l}^{n}-q_{i,l}^{n*}\right]+\sum_{k=1}^{O}\sum_{i=1}^{M}\left[-p_{k,i}^{s*}+\vartheta \varpi_{i}^{*}\right]\times \left[q_{k,i}^{r}-q_{k,i}^{r*}\right]+\sum_{i=1}^{M}\left[q_{i}^{v*}-\sum_{l=1}^{T}q_{i,l}^{n*}\right]\times \left[\sigma_{i}-\sigma_{i}^{*}\right]+\\ \sum_{i=1}^{M}\left[\vartheta \cdot \beta \sum_{l=1}^{T}q_{i,l}^{n*}-\sum_{k=1}^{O}q_{k,i}^{r*}\right]\times \left[\varpi_{i}-\varpi_{i}^{*}\right]\geq 0\\ \forall \left(Q^{v},Q^{1},Q^{7},\sigma ,\varpi \right)\in R_{+}^{M+MT+OM+2M}. \end{array}$$

*In VI (32), $\sigma_{i}$ and $\varpi_{i}$ are the multipliers of (29) and (30), respectively*.

**Proof.**  The proof can be obtained from that of Theorem 1. □

### 5.2. Equilibrium of the Retailers

When the OEMs choose the ARS, the T retailers in the network only need to purchase new products from the M OEMs and sell them to N demand markets to satisfy the demands of the consumers; they are not responsible for the trading of the remanufactured products. Suppose that during one OEM’s production period, the retailer l purchases $q_{i,l}^{n}$ items of new products from the OEM i with the endogenous price of $p_{i,l}^{n}$ per item; moreover, retailer l also needs to pay the total exhibition cost $c_{l}^{n}\left(Q^{1}\right)$. Furthermore, the retailer l sells $q_{l,j}^{n}$ new products to the demand market j with the endogenous price of $p_{l,j}^{n}$ per item. Thus, the net profit of the retailer l in this model, denoted by $\prod_{i}^{2}$, is as follows:

(33) $$\max\Pi_{l}^{2}=\sum_{j=1}^{N}p_{l,j}^{n}q_{l,j}^{n}-\sum_{i=1}^{M}p_{i,l}^{n}q_{i,l}^{n}-c_{l}^{n}\left(Q^{1}\right)$$

(34) $$s.t.\sum_{j=1}^{N}q_{l,j}^{n}\leq \sum_{i=1}^{M}q_{i,l}^{n}$$

(35) $$q_{i,l}^{n},q_{l,j}^{n}\geq 0,\;\forall j,\forall i$$

Constraint (34) indicates that the sales of new products between the retailer l and all the demand markets cannot exceed all the new products purchased by the retailer l from all the OEMs.

**Proposition 7.**  *In the ARS model, the retailer, the profit function of retailer is a concave function of its decision variables*.

**Proof.**  The proof is similar to of Proposition 1. □

**Theorem 6.**  Assume that in the ARS model, all the retailers are competing non-cooperatively. Then, from Proposition 7 and Assumption 7, the equilibrium of all retailers satisfies the VI as defined in (36). In other words, find $\left(Q^{1*},Q^{3*},\xi^{*}\right)\in R_{+}^{MT+TN+T}$ which satisfies the following VI:

(36) $$\begin{array}{l} \sum_{i=1}^{M}\sum_{l=1}^{T}\left[p_{i,l}^{n*}+\frac{\partial c_{l}^{n}\left(Q^{1*}\right)}{\partial q_{i,l}^{n}}-\xi_{l}^{*}\right]\times \left[q_{i,l}^{n}-q_{i,l}^{n*}\right]+\sum_{l=1}^{T}\sum_{j=1}^{N}\left[-p_{l,j}^{n*}+\xi_{l}^{*}\right]\times \left[q_{l,j}^{n}-q_{l,j}^{n*}\right]+\\ \sum_{l=1}^{T}\left[\sum_{i=1}^{M}q_{i,l}^{n*}-\sum_{j=1}^{N}q_{l,j}^{n*}\right]\times \left[\xi_{l}-\xi_{l}^{*}\right]\geq 0,\forall \left(Q^{1},Q^{3},\xi \right)\in R_{+}^{MT+TN+T}. \end{array}$$

*In VI (36), $\xi_{l}$ is the Lagrange multiplier of (34)*.

**Proof.**  The proof is similar to of Theorem 1. □

### 5.3. Equilibrium Decisions of the Demand Markets for New Products

When the OEMs choose the ARS, the consumers in each of the N demand markets can choose to purchase new products from the retailers or purchase remanufactured products from the 3PRs.

Considering the transaction of the new products in the demand market j of the ARS model, then from (14) and (15), we know that the price and the sales of the new products should satisfy the following CPs:

(37) $$p_{l,j}^{n}+c_{l,j}^{n}\left(q_{l,j}^{n}\right)\left\{ \begin{array}{l} =\rho_{j}^{n},q_{l,j}^{n}>0\\ \geq \rho_{j}^{n},q_{l,j}^{n}=0 \end{array}\right.$$

(38) $$d_{j}^{n}\left(\rho_{j}^{n},\rho_{j}^{r}\right)\left\{ \begin{array}{l} =\sum_{l=1}^{T}q_{l,j}^{n}\;\;if\;\rho_{j}^{n}>0\\ \leq \sum_{l=1}^{T}q_{l,j}^{n}\;\;if\;\rho_{j}^{n}=0 \end{array}\right.$$

In (38), the demand for new products $d_{j}^{n}\left(\rho_{j}^{n},\rho_{j}^{r}\right)$ should satisfy (1). The explanations of equations (37) and (38) are the same as those given for (14) and (15).

When the OEMs choose the ARS, the used products in the demand markets are collected by the O 3PRs for remanufacturing. Since only new products can be recycled for further use and remanufactured products cannot be further recycled, the activities of the reverse logistics in the CLSC network apply to new products only. Thus, after the new products have been consumed by the consumers and become used products, they are recycled and remanufactured by the O 3PRs. Hence, if the consumers from the demand market j are selling their used products to the demand market j and purchasing remanufactured products from the 3PRs via the demand market j, then the used products returned to 3PR k from the demand market j should satisfy the following CPs:

(39) $$a_{j}\left(\sum_{k=1}^{O}q_{j,k}^{e}\right)\left\{ \begin{array}{l} =\rho_{j}^{e},\;if\;q_{j,k}^{e}>0\\ \geq \rho_{j}^{e},\;if\;q_{j,k}^{e}=0 \end{array}\right.\;$$

(40) $$s.t.\sum_{k=1}^{O}q_{j,k}^{e}\leq \sum_{l=1}^{T}q_{l.j}^{n}$$

(41) $$q_{l,j}^{n},q_{j,k}^{e}\geq 0,\;\forall l,\;\;\forall k$$

Constraint (40) indicates that the used products returned from the demand market j to all the 3PRs for recycling should not exceed the total new products sold to the demand market by all the retailers.

The equilibrium of the demand markets for the new products can be obtained by using the equivalence between CP and VI [46]:

**Theorem 7.**  In the ARS model, the equilibrium of all the demand markets for the new product is obtained by solving the following VI: Find $\left(Q^{3*},\rho^{n*},Q^{6*},\theta^{*}\right)\in R_{+}^{TN+N+NO+N}$ which satisfies

(42) $$\begin{array}{l} \sum_{l=1}^{T}\sum_{j=1}^{N}\left[p_{l,j}^{n*}+c_{l,j}^{n}\left(q_{l,j}^{n*}\right)-\rho_{j}^{n*}-\theta_{j}^{*}\right]\times \left[q_{l,j}^{n}-q_{l,j}^{n*}\right]+\\ \sum_{j=1}^{N}\left[\sum_{l=1}^{T}q_{l,j}^{n*}-d_{j}^{n}\left(\rho_{j}^{n*},\rho_{j}^{r*}\right)\right]\times \left[\rho_{j}^{n}-\rho_{j}^{n*}\right]+\\ \sum_{j=1}^{N}\sum_{k=1}^{O}\left[a_{j}\left(\sum_{k=1}^{O}q_{j,k}^{e*}\right)-\rho_{j}^{e*}+\theta_{j}^{*}\right]\times \left[q_{j,k}^{e}-q_{j,k}^{e*}\right]+\\ \sum_{j=1}^{N}\left[\sum_{l=1}^{T}q_{l.j}^{n*}-\sum_{k=1}^{O}q_{j,k}^{e*}\right]\times \left[\theta_{j}-\theta_{j}^{*}\right]\geq 0,\forall \left(Q^{3},\rho^{n},Q^{6},\theta \right)\in R_{+}^{TN+N+NO+N}. \end{array}$$

*In VI (42), $\theta_{j}$ is the Lagrange multiplier of (40)*.

### 5.4. Equilibrium of the 3PRs

When the OEMs choose the ARS, the 3PRs in the CLSC Network are authorized by the OEMs to recycle the used products. Thus, the 3PRs are responsible for the payments of all the costs associated with the production and the trading of the remanufactured products to satisfy the demand of the green consumers. Suppose that during one production cycle of the OEMs, the 3PR k collects $q_{j,k}^{e}$ items of the used products from the demand market j via the OEMs. He/she needs to pay the basic costs, which include a transaction cost $c_{j,k}^{e}\left(q_{j,k}^{e}\right)$, a collection cost $\rho_{j}^{e}q_{j,k}^{e}$, and an endogenous price $\rho_{j}^{e}$ per item for the purchase of used products. Moreover, after the used products have been returned by the demand markets to the OEMs, they must thoroughly inspect these used products before they can authorize the 3PRs for remanufacturing. Thus, the 3PR k also needs to pay the total inspection cost $\phi_{k}\left(\sum_{j=1}^{N}q_{j,k}^{e}\right)$ for the inspection of the used products. After thoroughly inspecting, only $\beta \sum_{j=1}^{N}q_{j,k}^{e}$ items of the used products can be remanufactured. For the remaining $\bar{\beta }\sum_{j=1}^{N}q_{j,k}^{e}$ items (where $\bar{\beta }=1-\beta$) that cannot be remanufactured, they need to be sent to the landfill for disposal. Thus, the 3PR k also needs to pay the total transportation cost $c_{k,D}\left(\bar{\beta }\sum_{j=1}^{N}q_{j,k}^{e}\right)$ for transferring the waste products to the landfill site, and the landfill cost $\bar{\beta }\bar{\rho }\sum_{j=1}^{N}q_{j,k}^{e}$ for the disposal of all the waste products at the landfill. After the 3PR k has been authorized by the OEMs to recycle the used products, he/she needs to remanufacture these used products with the production cost $r_{k}\left(\sum_{i=1}^{M}q_{k,i}^{r},\chi \right)$, where χ represents the 3PR‘s green innovation effort and $\frac{\partial r_{k}\left(\sum_{i=1}^{M}q_{k,i}^{r},\chi \right)}{\partial \chi }<0$, which indicates the higher the OEMs’ green innovation effort, the lower the production cost of the new products. After the used products have been manufactured by the 3PR k, he/she sells $q_{k,j}^{r}$ items of these remanufactured products to the demand market j with the endogenous price of $p_{k,j}^{r}$ per item. However, he/she needs to pay the transaction cost $c_{k,j}^{r}\left(q_{k,j}^{r}\right)$ for selling these remanufactured products to the demand market j and pays the patent licensing fees $\sum_{i=1}^{M}p_{k,i}^{s}q_{k,i}^{r}$ to all the OEMs, where $p_{k,i}^{s}$ is the patent licensing fee per item. From the carbon emission and trading system for enterprises implemented by the government described in Section 4, we know that when the carbon emission from the production of the remanufactured products of the 3PR k in one production cycle exceeds the upper limit $\bar{M}$, he/she needs to pay the carbon emission costs of $v\left(e_{r}\sum_{i=1}^{M}q_{k,i}^{r}-\bar{M}\right)$ to produce the remanufactured products, where ν is the unit price of carbon trading, $e_{r}$ is the environmental impact of one item of the remanufactured products, and $e_{r}\sum_{i=1}^{M}q_{k,i}^{r}$ is the amount of carbon emission from the production of the remanufactured products of the 3PR k in one production cycle. This carbon emission and trading system for enterprises is consistent with that given in the life cycle assessment (LCA) [45].

Thus, the income of the 3PR k in the ARS model includes that obtained by selling remanufactured products to the N markets, whereas his/her costs include the production cost, the recycling cost, the carbon emission under the carbon trading system, and the patent incensing fee. Thus, the net profit of the 3PR k in the ARS model, denoted by $\prod_{k}^{2}$, can be expressed as follows:

(43) $$\begin{array}{l} \max\Pi_{k}^{2}=\sum_{j=1}^{N}p_{k,j}^{r}q_{k,j}^{r}-\sum_{j=1}^{N}c_{k,j}^{r}\left(q_{k,j}^{r}\right)-\sum_{j=1}^{N}\rho_{j}^{e}q_{j,k}^{e}-c_{j,k}^{e}\left(q_{j,k}^{e}\right)\\ -\phi_{k}\left(\sum_{j=1}^{N}q_{j,k}^{e}\right)-c_{k,D}\left(\bar{\beta }\sum_{j=1}^{N}q_{j,k}^{e}\right)-\bar{\beta }\bar{\rho }\sum_{j=1}^{N}q_{j,k}^{e}-r_{k}\left(\sum_{i=1}^{M}q_{k,i}^{r},\chi \right)\\ -\sum_{i=1}^{M}p_{k,i}^{s}q_{k,i}^{r}-v\left(e_{r}\sum_{i=1}^{M}q_{k,i}^{r}-\bar{M}\right) \end{array}$$

(44) $$s.t.\sum_{i=1}^{M}q_{k,i}^{r}\leq \beta \sum_{j=1}^{N}q_{j,k}^{e}$$

(45) $$\sum_{j=1}^{N}q_{k,j}^{r}\leq \sum_{i=1}^{M}q_{k,i}^{r}$$

(46) $$q_{j,k}^{e},q_{k,i}^{r},q_{k,j}^{r}\geq 0,\;\forall j,\;\forall i$$

Constraint (44) indicates that the sales of all products of the 3PR k authorized by all the OEMs for remanufacturing should not exceed the usable amount of used products recovered from all the demand markets. Constraint (45) indicates that the sales of all green remanufactured products of the 3PR k sold to all the N demand markets should not exceed his/her total remanufactured quantity.

**Proposition 8.**  *In the ARS model, the profit function of 3PR k is concave*.

**Proof.**  The proof of this theorem is similar to of Proposition 1. □

**Theorem 8.**  Assume that in the ARS model, all the 3PRs are competing in a non-cooperative manner. Then, from Proposition 8 and Assumption 7, the equilibrium of all 3PRs satisfies the VI as defined in (47). In other words, find $\left(Q^{6*},Q^{7*},Q^{8*},\kappa^{*},\psi^{*}\right)\in R_{+}^{NO+ON+OM+2O}$ which satisfies the following VI:

(47) $$\begin{array}{l} \sum_{j=1}^{N}\sum_{k=1}^{O}\left[\rho_{j}^{e*}+\frac{\partial \phi_{k}\left(\sum_{j=1}^{N}q_{j,k}^{e*}\right)}{\partial q_{j,k}^{e}}+\overset{-}{\beta }\overset{-}{\rho }+\frac{\partial c_{k,D}\left(\bar{\beta }\sum_{j=1}^{N}q_{j,k}^{e*}\right)}{\partial q_{j,k}^{e}}+\frac{\partial c_{j,k}^{e}\left(q_{j,k}^{e}\right)}{\partial q_{j,k}^{e}}-\beta \kappa_{k}^{*}\right]\times \left[q_{j,k}^{e}-q_{j,k}^{e*}\right]\\ +\sum_{k=1}^{O}\sum_{j=1}^{N}\left[-p_{k,j}^{r*}+\frac{\partial c_{k,j}^{r}\left(q_{k,j}^{r*}\right)}{\partial q_{k,j}^{r}}+\psi_{k}^{*}\right]\times \left[q_{k,j}^{r}-q_{k,j}^{r*}\right]\\ +\sum_{k=1}^{O}\sum_{i=1}^{M}\left[\frac{\partial r_{k}\left(\sum_{i=1}^{M}q_{k,i}^{r*},\chi \right)}{\partial q_{k,i}^{r}}+p_{k,i}^{s*}-\psi_{k}^{*}+\kappa_{k}^{*}+ve_{r}\right]\times \left[q_{k,i}^{r}-q_{k,i}^{r*}\right]\\ +\sum_{k=1}^{O}\left[\beta \sum_{j=1}^{N}q_{j,k}^{e*}-\sum_{i=1}^{M}q_{k,i}^{r*}\right]\times \left[\kappa_{k}-\kappa_{k}^{*}\right]+\sum_{k=1}^{O}\left[\sum_{i=1}^{M}q_{k,i}^{r*}-\sum_{j=1}^{N}q_{k,j}^{r*}\right]\times \left[\psi_{k}-\psi_{k}^{*}\right]\geq 0,\\ \forall \left(Q^{6},Q^{7},Q^{8},\kappa ,\psi \right)\in R_{+}^{NO+ON+OM+2O}. \end{array}$$

*In VI (47), $\kappa_{k}$ and $\psi_{k}$ are the Lagrange multipliers of (44) and (45), respectively*.

**Proof.**  The proof is similar to of Theorem 1. □

### 5.5. Equilibrium Decisions of the Demand Markets for Remanufactured Products

When the OEMs choose the ARS, the O 3PRs are selling green remanufactured products to the N demand markets. Considering the transactions of remanufactured products in the demand market j of the ARS model, then from (19) and (20), we know that the price of the remanufactured products and the demand for the remanufactured products $\rho_{j}^{r}$ should satisfy the following CPs:

(48) $$p_{k,j}^{r}+c_{k,j}^{r}\left(q_{k,j}^{r}\right)\left\{ \begin{array}{l} =\rho_{j}^{r},q_{k,j}^{r}>0\\ \geq \rho_{j}^{r},q_{k,j}^{r}=0 \end{array}\right.$$

(49) $$d_{j}^{r}\left(\rho_{j}^{n},\rho_{j}^{r}\right)\left\{ \begin{array}{l} =\sum_{k=1}^{O}q_{k,j}^{r}\;\;if\;\rho_{j}^{r}>0\\ \leq \sum_{k=1}^{O}q_{k,j}^{r}\;\;if\;\rho_{j}^{r}=0 \end{array}\right.$$

In (49), the demand for green manufactured products $d_{j}^{r}\left(\rho_{j}^{n},\rho_{j}^{r}\right)$ should satisfy (2). CP (48) indicates that if the green consumers from the demand market j are willing to purchase remanufactured products from the 3PR k, then the price they need to pay for these remanufactured products, $\rho_{j}^{r}$, is equal to the sum of the purchase cost and the transaction cost for the transaction cost of these remanufactured products between retailer the l and the demand market j. CP (49) indicates that the demand for remanufactured products from the green consumers in the demand market j is equal to the total remanufactured products purchased by the demand market j from all the 3PRs.

The equilibrium of the demand markets for the remanufactured products can be obtained by using the equivalence between CP and VI [46].

**Theorem 9.**  In the ARS model, the equilibrium of all the demand markets for the remanufactured products is obtained by solving the following VI: Find $\left(Q^{8*},\rho^{r*}\right)\in R_{+}^{ON+N}$ which satisfies the following VI:

(50) $$\begin{array}{l} \sum_{k=1}^{O}\sum_{j=1}^{N}\left[p_{k,j}^{r*}+c_{k,j}^{r}\left(q_{k,j}^{r*}\right)-\rho_{j}^{r*}\right]\times \left[q_{k,j}^{r}-q_{k,j}^{r*}\right]+\\ \sum_{j=1}^{N}\left[\sum_{k=1}^{T}q_{k,j}^{r*}-d_{j}^{r}\left(\rho_{j}^{n*},\rho_{j}^{r*}\right)\right]\times \left[\rho_{j}^{r}-\rho_{j}^{r*}\right]\geq 0,\forall \left(Q^{8},\rho^{r}\right)\in R_{+}^{ON+N}. \end{array}$$

### 5.6. Equilibrium Decisions of the Network in the ARS Model

Suppose the decisions of all the decision-makers are at equilibrium simultaneously, then VI (32), (36), (39), (44), and (50) hold simultaneously. By adding these VIs together and subtracting all the endogenous prices ($p_{i,l}^{n}$, $p_{k,i}^{s}$, $p_{l,j}^{n}$, $p_{k,j}^{r}$, and $\rho_{j}^{e}$), the equilibrium of the network is obtained as follows:

**Theorem 10.**  In the ARS model, the equilibrium of the network is obtained from the following VI: Find $\left(Q^{v*},Q^{1*},Q^{3*},Q^{6*},Q^{7*},Q^{8*},\rho^{n*},\rho^{r*},\sigma^{*},\varpi^{*},\xi^{*},\theta^{*},\kappa^{*},\mu^{*}\right)\in R_{+}^{M+MT+TN+NO+OM+ON+2N+2M+T+N+2O}$ which satisfies the following VI:

(51) $$\begin{array}{l} \sum_{i=1}^{M}\left[\frac{\partial f_{i}\left(Q^{v*},\chi \right)}{\partial q_{i}^{v}}-\sigma_{i}^{*}+ve_{n}\right]\times \left[q_{i}^{v}-q_{i}^{v*}\right]+\\ \sum_{i=1}^{M}\sum_{l=1}^{T}\left[\frac{\partial c_{i,l}^{n}\left(q_{i,l}^{n*}\right)}{\partial q_{i,l}^{n}}+\sigma_{i}^{*}-\beta \varpi_{i}^{*}+\frac{\partial c_{l}^{n}\left(Q^{1*}\right)}{\partial q_{i,l}^{n}}-\xi_{l}^{*}\right]\times \left[q_{i,l}^{n}-q_{i,l}^{n*}\right]+\\ \sum_{l=1}^{T}\sum_{j=1}^{N}\left[\xi_{l}^{*}+c_{l,j}^{n}\left(q_{l,j}^{n*}\right)-\rho_{j}^{n*}-\theta_{j}^{*}\right]\times \left[q_{l,j}^{n}-q_{l,j}^{n*}\right]+\\ \sum_{j=1}^{N}\sum_{k=1}^{O}\left[a_{j}\left(\sum_{k=1}^{O}q_{j,k}^{e*}\right)+\theta_{j}^{*}+\frac{\partial \phi_{k}\left(\sum_{j=1}^{N}q_{j,k}^{e*}\right)}{\partial q_{j,k}^{e}}+\overset{-}{\beta }\overset{-}{\rho }+\frac{\partial c_{k,D}\left(\bar{\beta }\sum_{j=1}^{N}q_{j,k}^{e*}\right)}{\partial q_{j,k}^{e}}+\frac{\partial c_{j,k}^{e}\left(q_{j,k}^{e*}\right)}{\partial q_{j,k}^{e}}-\beta \kappa_{k}^{*}\right]\times \\ \left[q_{j,k}^{e}-q_{j,k}^{e*}\right]+\sum_{k=1}^{O}\sum_{i=1}^{M}\left[\frac{\partial r_{k}\left(\sum_{i=1}^{M}q_{k,i}^{r*},\chi \right)}{\partial q_{k,i}^{r}}-\psi_{k}^{*}+\kappa_{k}^{*}+ve_{r}+\vartheta \varpi_{i}^{*}\right]\times \left[q_{k,i}^{r}-q_{k,i}^{r*}\right]\\ +\sum_{k=1}^{O}\sum_{j=1}^{N}\left[\frac{\partial c_{k,j}^{r}\left(q_{k,j}^{r*}\right)}{\partial q_{k,j}^{r}}+\psi_{k}^{*}+c_{k,j}^{r}\left(q_{k,j}^{r*}\right)-\rho_{j}^{r*}\right]\times \left[q_{k,j}^{r}-q_{k,j}^{r*}\right]+\sum_{j=1}^{N}\left[\sum_{l=1}^{T}q_{l,j}^{n*}-d_{j}^{n}\left(\rho_{j}^{n*},\rho_{j}^{r*}\right)\right]\times \\ \left[\rho_{j}^{n}-\rho_{j}^{n*}\right]+\sum_{j=1}^{N}\left[\sum_{k=1}^{T}q_{k,j}^{r*}-d_{j}^{r}\left(\rho_{j}^{n*},\rho_{j}^{r*}\right)\right]\times \left[\rho_{j}^{r}-\rho_{j}^{r*}\right]+\sum_{i=1}^{M}\left[q_{i}^{v*}-\sum_{l=1}^{T}q_{i,l}^{n*}\right]\times \left[\sigma_{i}-\sigma_{i}^{*}\right]+\\ \sum_{i=1}^{M}\left[\vartheta \cdot \beta \sum_{l=1}^{T}q_{i,l}^{n*}-\sum_{k=1}^{O}q_{k,i}^{r*}\right]\times \left[\varpi_{i}-\varpi_{i}^{*}\right]+\sum_{l=1}^{T}\left[\sum_{i=1}^{M}q_{i,l}^{n*}-\sum_{j=1}^{N}q_{l,j}^{n*}\right]\times \left[\xi_{l}-\xi_{l}^{*}\right]+\\ \sum_{j=1}^{N}\left[\sum_{l=1}^{T}q_{l.j}^{n*}-\sum_{k=1}^{O}q_{j,k}^{e*}\right]\times \left[\theta_{j}-\theta_{j}^{*}\right]+\sum_{k=1}^{O}\left[\beta \sum_{j=1}^{N}q_{j,k}^{e*}-\sum_{i=1}^{M}q_{k,i}^{r*}\right]\times \left[\kappa_{k}-\kappa_{k}^{*}\right]+\\ \sum_{k=1}^{O}\left[\sum_{i=1}^{M}q_{k,i}^{r*}-\sum_{j=1}^{N}q_{k,j}^{r*}\right]\times \left[\psi_{k}-\psi_{k}^{*}\right]\geq 0\\ \forall \left(Q^{v},Q^{1},Q^{3},Q^{6},Q^{7},Q^{8},\rho^{n},\rho^{r},\sigma ,\varpi ,\xi ,\theta ,\kappa ,\psi \right)\in R_{+}^{M+MT+TN+NO+OM+ON+2N+2M+T+N+2O}. \end{array}$$

The existence and uniqueness of VI (51) is obtained from the fact that the functions in VI (51) are monotone and Lipschitz continuous [14].

From the equivalence of VI (32) and CP [46], the endogenous price of new products $p_{i,l}^{n*}$ and the patent licensing fee $p_{k,i}^{s*}$ at equilibrium are as follows:

(52) $$p_{i,l}^{n*}=\frac{\partial c_{{}_{i,l}}^{n}\left(q_{i,l}^{n*}\right)}{\partial q_{i,l}^{n}}+\sigma_{i}^{*}-\beta \varpi_{i}^{*}$$

(53) $$p_{k,i}^{s*}=\vartheta \varpi_{i}^{*}.$$

Similarly,

(54) $$p_{l,j}^{n*}=\xi_{l}^{*}.$$

(55) $$p_{k,j}^{r*}=\frac{\partial c_{k,j}^{r}\left(q_{k,j}^{r*}\right)}{\partial q_{k,j}^{r}}+\mu_{k}^{*}.$$

(56) $$\rho_{j}^{e*}=a_{j}\left(\sum_{k=1}^{O}q_{j,k}^{e*}\right)+\theta_{j}^{*}.$$

**Proposition 9.**  *In the ARS model, the decision variables, i.e., $q_{i}^{v}$, $q_{i,l}^{n}$, $q_{j,k}^{e}$, and $q_{k,j}^{r}$, are decreasing functions of the parameter ν, where ν is the unit price of carbon trading fixed by the government*.

**Proof.**  See Appendix D. □

**The Economic Explanation of Proposition 9.**  As in the IHRS model, when the OEMs reduce the production of new products, resulting in a reduction in the amount of recycling, the number of remanufactured products in the CLSC network also decreases.

**Proposition 10.**  *In the ARS model, the decision variables, i.e., $q_{i}^{v}$, $q_{i,l}^{n}$, $q_{j,k}^{e}$, and $q_{k,j}^{r}$, are increasing functions of the parameter χ, where χ is the OEMs’ green innovation efforts*.

**Proof.**  The proof is similar to of Proposition 3. □

**The Economic Explanation of Proposition 10.**  As in the IHRS model, green innovation efforts develop a good reputation for the OEMs and the 3PRs, increasing consumers’ willingness to buy companies’ products; consequently, consumers’ demand for products increases, forming a virtuous circle in the CLSC.

**Proposition 11.**  *In the ARS model, the decision variables, i.e., $q_{i}^{v}$, $q_{i,l}^{n}$, $q_{j,k}^{e}$, and $q_{k,j}^{r}$, are decreasing functions of the parameter τ, where τ is the proportion of the green consumers in the markets*.

**Proof.**  The proof is similar to of Proposition 9. □

**The Economic Explanation of Proposition 11.**  As in the IHRS model, when the 3PRs pay attention to the production of the remanufactured products, a network for remanufactured products will be formed, and the demands for the two products will be decreased.

## 6. Numerical Examples

In this section, we use a numerical example to illustrate how the following parameters can affect the equilibrium policies and the profits of the decision-maker: the unit carbon trading price, the green innovation effort, and the proportion of green consumers. In this numerical example, we consider that there are 2 OEMs, 2 retailers, and 2 demand markets in the network of the IHRS, and there are 2 OEMs, 2 retailers, 2 demand markets, and 2 3PRs in the network of the ARS. (That is, i=1,2, l=1,2, and j=1,2 in Figure 1 and i=1,2, l=1,2, j=1,2, and k=1,2 in Figure 2.) The costs of the decision-makers [12] are as follows:

Costs of the OEMs:

(57) $$f_{i}\left(Q^{v},\chi \right)=\left(2.5q_{i}^{v}+q_{1}^{v}q_{2}^{v}+2q_{i}^{v}\right)+0.5\chi q_{i}^{v},i=1,2$$

(58) $$c_{i,l}^{n}\left(q_{i,l}^{n}\right)=0.1{\left(q_{i,l}^{n}\right)}^{2}+q_{i,l}^{n},i=1,2\;l=1,2$$

(59) $$c_{j,i}^{e}\left(q_{j,i}^{e}\right)=q_{j,i}^{e}+5,\;i=1,2\;j=1,2$$

(60) $$\phi_{i}\left(\sum_{j=1}^{N}q_{j,i}^{e}\right)=1.8\sum_{j=1}^{N}q_{j,i}^{e}+3,\;i=1,2\;$$

(61) $$c_{i,D}\left(\bar{\beta }\sum_{j=1}^{N}q_{j,i}^{e}\right)=0.5{\left(\bar{\beta }\sum_{j=1}^{N}q_{j,i}^{e}\right)}^{2}+3.5\bar{\beta }\sum_{j=1}^{N}q_{j,i}^{e},i=1,2$$

(62) $$c_{i,l}^{r}\left(q_{i,l}^{r}\right)=q_{i,l}^{r}+5,i=1,2\;l=1,2$$

(63) $$r_{i}\left(\sum_{l}^{T}q_{i,l}^{r},\chi \right)={\left(\sum_{l=1}^{T}q_{i,l}^{r}\right)}^{2}+\sum_{l=1}^{T}q_{i,l}^{r}+0.5\left(1-\chi \right)3.5\sum_{l=1}^{T}q_{i,l}^{r},i=1,2\;$$

Costs of the retailers:

(64) $$c_{l}^{n}\left(Q^{1}\right)=0.5{\left(\sum_{i=1}^{M}q_{i,l}^{n}\right)}^{2},\;l=1,2$$

(65) $$c_{l}^{r}\left(Q^{2}\right)=0.5{\left(\sum_{i=1}^{M}q_{i,l}^{r}\right)}^{2},\;l=1,2$$

Costs of the demand markets:

(66) $$a_{j}\left(\sum_{i=1}^{M}q_{j,i}^{e}\right)=0.5{\left(\sum_{i=1}^{M}q_{j,i}^{e}\right)}^{2},\;j=1,2\;$$

(67) $$a_{j}\left(\sum_{i=1}^{M}q_{j,k}^{e}\right)=0.5{\left(\sum_{i=1}^{M}q_{j,k}^{e}\right)}^{2},\;j=1,2\;$$

Cost functions of the 3PRs:

(68) $$c_{j,k}^{e}\left(q_{j,k}^{e}\right)=q_{j,k}^{e}+5\;j=1,2\;k=1,2$$

(69) $$\phi_{k}\left(\sum_{j=1}^{N}q_{j,k}^{e}\right)=1.8\sum_{j=1}^{N}q_{j,k}^{e}+3,k=1,2$$

(70) $$c_{k,D}\left(\bar{\beta }\sum_{j=1}^{N}q_{j,k}^{e}\right)=0.5{\left(\bar{\beta }\sum_{j=1}^{N}q_{j,k}^{e}\right)}^{2}+3.5\bar{\beta }\sum_{j=1}^{N}q_{j,k}^{e},j=1,2\;$$

(71) $$r_{k}\left(\sum_{i}^{M}q_{k,i}^{r},\chi \right)={\left(\sum_{i}^{M}q_{k,i}^{r}\right)}^{2}+\sum_{i}^{M}q_{k,i}^{r}+0.5\left(1-\chi \right)3.5\sum_{i}^{M}q_{k,i}^{r},k=1,2\;$$

(72) $$c_{k,j}^{r}\left(q_{k,j}^{r}\right)=q_{k,j}^{r}+5\;k=1,2\;j=1,2$$

The above cost functions and demand functions ensure that the monotone and Lipschitz continuity of VI (22) and VI (51) hold. Therefore, when the OEMs choose the IHRS, we use the modified projection algorithm [47], which is compiled by MATLAB, to solve VI (22). The profits of the OEMs and the retailers are obtained by (3) and (9), respectively. When the OEMs choose the ARS, we solve VI (51) to obtain the profits of the OEMS, the retailers, and the 3PRs given by (28), (33), and (43), respectively. The profit of the network in IHRS is equal to the sum of the profits of the OEMs and retailers, and the profit of the network in ARS is equal to the sum of the profits of the OEMs, the retailers, and the 3PRs. We chose the step size of each iteration of the modified projection algorithm to be 0.03 and the termination error to be $10^{-7}$ and the initial value of each decision variable to be 1.

### 6.1. The Impact of the Unit Carbon Trading Price

This section mainly analyzes the impact of the unit carbon trading price charged by the government on the sales quantities, the prices of the products, the profit of each decision-maker, and the choice of the OEMs’ remanufacturing strategies. Since the lower limit of the recovery rate of the used products can also affect the number of new products and remanufactured products in the demand markets, it can ultimately affect the choice of the OEMs’ remanufacturing strategies. Therefore, we analyze the effect of the unit carbon trading price on the decision variables and profits by using different lower limits of the recovering rates of the used products α: Case 1: a<0.7 (we use α=0.4 for illustrative purposes); Case 2: α>0.7 (we use α=0.7 for illustrative purposes). In each of the two cases, the other parameter settings are the same, i.e., β=0.9, $\bar{\rho }=0.5$, χ=0.5, τ=0.5, δ=0.8, $e_{n}=2$, $e_{r}=1.5$, and $\bar{M}=10$. We obtain the equilibrium, the carbon emission costs, and the profit of each decision-maker in both the IHRS and the ARS corresponding to different unit carbon trading price ν, where ν varies between 5 and 50, as follows:

Case 1: Low lower limit of the recovery rate (α=0.4).

**Figure 5:**
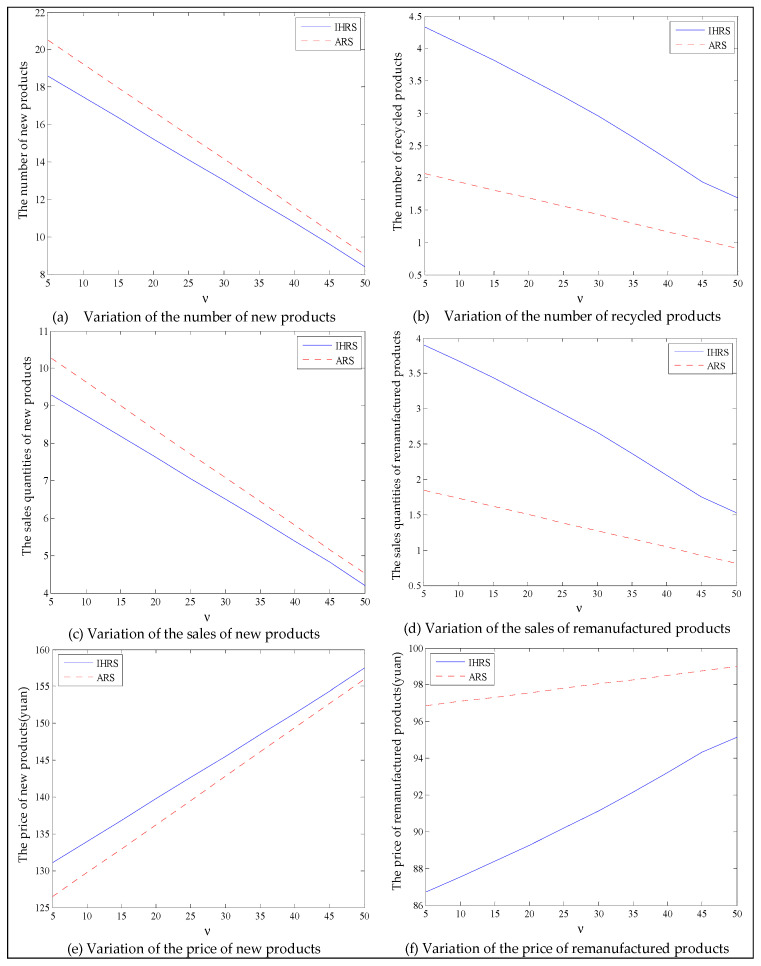
The variation of the equilibrium strategies in Case 1 of Section 6.1.

**Figure 6:**
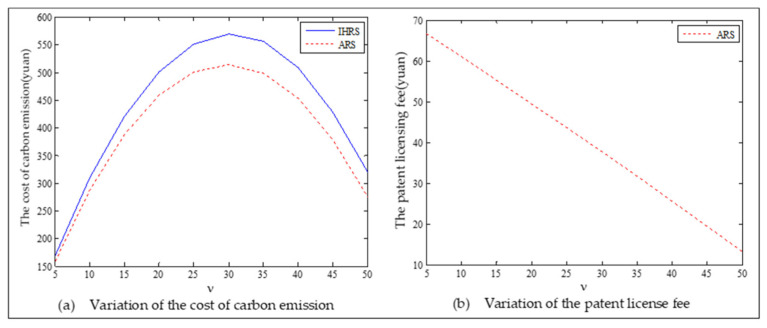
Changes of the costs of carbon emission and the patent licensing fee in Case 1 of Section 6.1.

**Figure 7:**
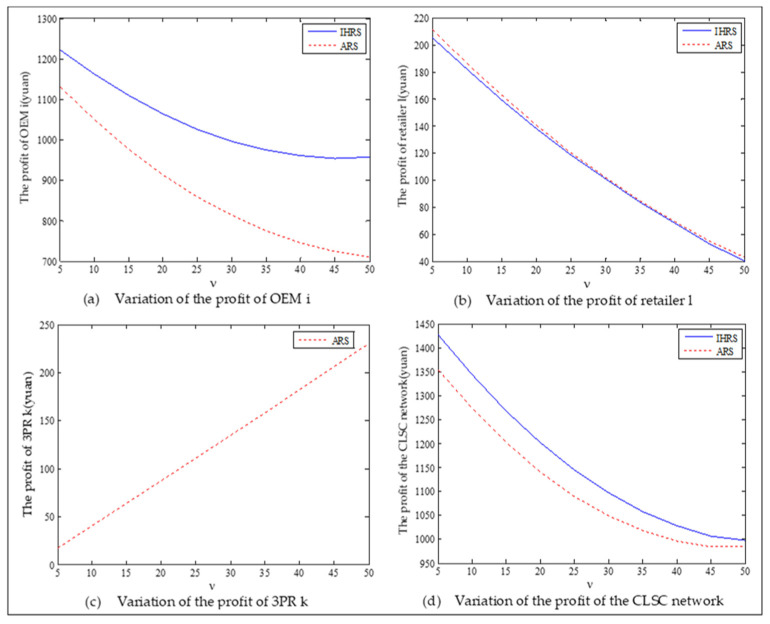
The variation of the profits in Case 1 of Section 6.1.

The equilibrium of the decision-makers is shown in Figure 5, the carbon emission costs due to the production of these two products of the IHRS model and the ARS model, and the patent licensing fee are shown in Figure 6, the profit of each decision-maker and that of the CLSC network are shown in Figure 7.

When the lower limit of the recovery rate is low, we observe from Figure 5 that when the unit carbon trading cost increases, the production quantities and the recycling quantities of the new products of both the IHRS model and the ARS model decrease; the sales quantities of these two products of both the IHRS model and the ARS model decrease, but their prices increase. The above directions of the movement of the decision variables with respect to the unit carbon trading cost are consistent with the statements of Proposition 3 and Proposition 9. Moreover, for Case 1, when the OEMs choose the IHRS, the sales quantities of the new products are lower than when they choose the ARS, but their prices are higher than when they choose the ARS; however, the differences in sale quantities and prices are not very significant. On the other hand, for Case 1, when the OEMs choose the IHRS, the quantities of the recycling products and the sale quantities of the remanufacturing products are higher than when they choose the ARS.

For Case 1, we observe from Figure 6 that when the unit carbon trading cost increases, the carbon emission costs charged to the OEMs and the 3PRs to produce these two products first increase and then decrease; the patent licensing fees paid by the 3PRs to the OEMs decrease.

For Case 1, from Figure 7, when the unit carbon trading cost increases, the profits of the OEMs, the retailers, and the entire CLSC network decrease, but the profits of the 3PRs increase; however, the differences in profit for each decision-maker between the IHRS and the ARS are not significant.

For Case 1, when the unit carbon trading price charged by the government increases and the lower limit of the recovery rate is low, fewer products are cycled for remanufacturing; thus, new products occupy most of the markets and the environmental impact of the new products is higher than that of the remanufactured products. When the unit carbon trading price increases, to avoid excessive carbon emission costs, OEMs will reduce their production of the new products, resulting in a reduction in the number of new products and the amount of recycling in the CLSC. Therefore, the number of remanufactured products also decreases and the prices of the two products rise due to the relationship between supply and demand. At the same time, because carbon emission costs first increase and then decrease, the increase in the carbon emission costs are much smaller than the decrease in revenue due to the changes in the quantity of the sales products, and both these two factors lead to a decrease in the profits of the OEMs. Since the 3PRs have less demand, they will also deliberately reduce the production of remanufactured products to reduce the carbon emissions; thus, the 3PRs will sell the excess carbon emissions allowances to other enterprises to obtain more profits.

Case 2: High lower limit of the recovery rate (α=1).

**Figure 8:**
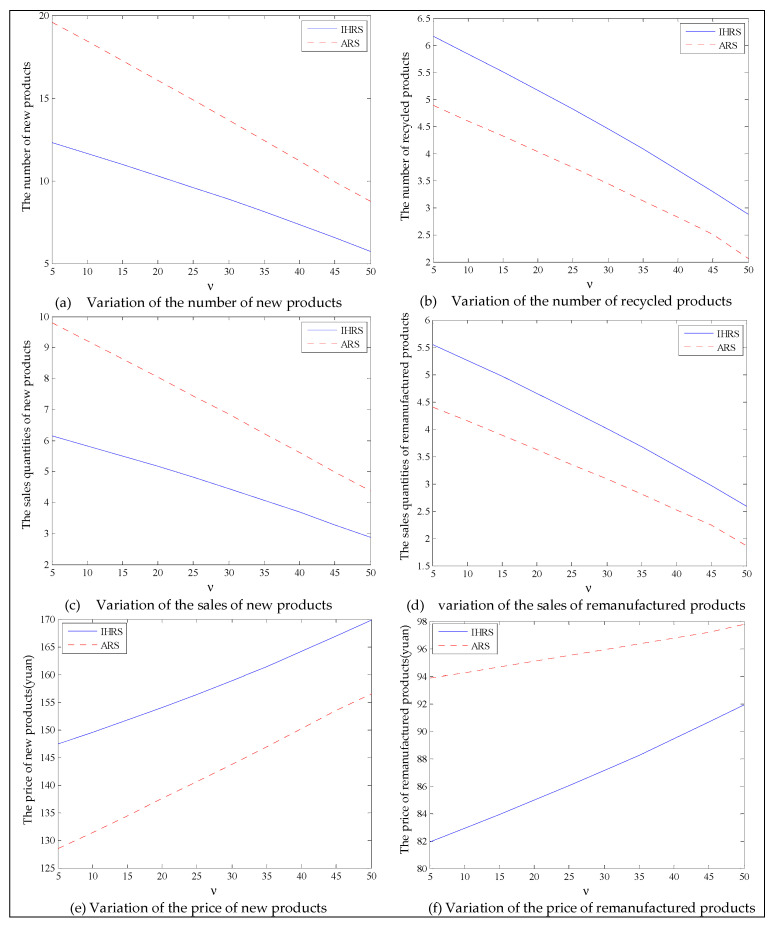
The variation of equilibrium strategies in Case 2 of Section 6.1.

**Figure 9:**
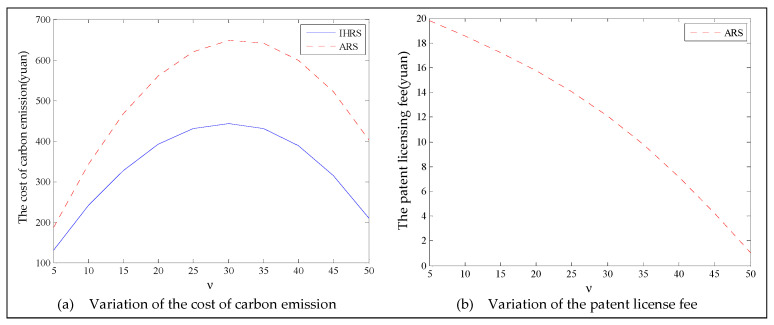
Changes in the costs of carbon emission and the patent licensing fee in Case 2 of Section 6.1.

**Figure 10:**
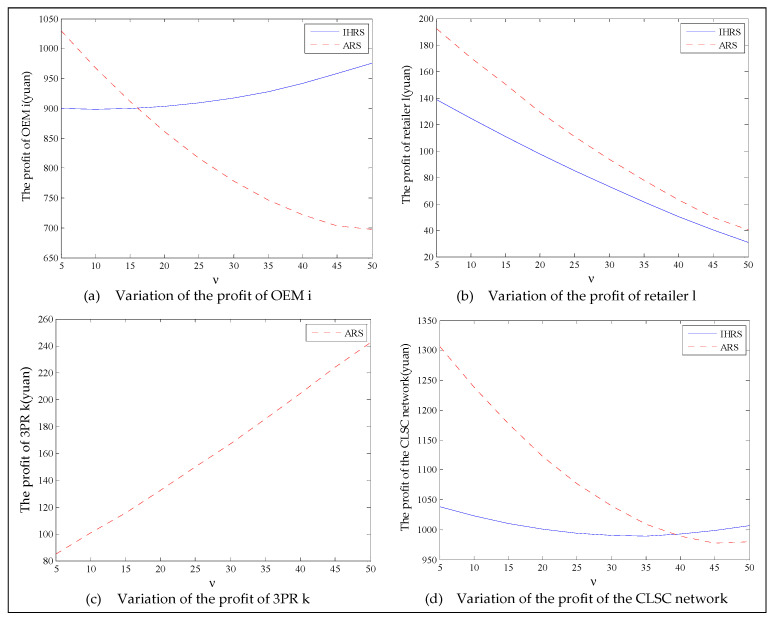
The variation of the profits in Case 2 of Section 6.1.

The equilibrium of the decision-makers is shown in Figure 8, the carbon emission costs due to the production of these two products of the IHRS model and the ARS model, and the patent licensing fee are shown in Figure 9, the profit of each decision-maker and that of the CLSC Network are shown in Figure 10.

When the lower limit of the recycling rate is high, from Figure 8, the directions of movement for all the decision variables in both models with respect to the unit carbon trading cost are the same as those for Case 1, which is consistent with the statements of Proposition 3 and Proposition 9. However, when the lower limit of the recovery rate increases, the differences in the production quantities, the sale, and the price of new products between the IHRS model and the ARS model become significant, and the sales quantities of the new products are larger than those of the remanufactured products when the OEMs choose the ARS. From Figure 10, in both the IHRS model and the ARS model of Case 2, when the unit carbon trading price is lower than 15, the profits of the OEMs when they choose the ARS are significantly larger than when they choose the IHRS; however, when the carbon trading price is higher than 15, the situation is reversed.

When the recovery rate is high, there are more remanufactured products being sold and consumers have more freedom in purchasing products, but the demand for these two products decrease. Therefore, the amount of carbon emissions due to production decreases, causing the OEMs to sell their excess carbon emission allowances to other enterprises to obtain more profits; on the other hand, due to the decrease in demand for the remanufactured products, the OEMs pay less for the carbon emission costs arising from the production of remanufactured products and hence their profits are increased.

When the unit carbon trading price is high, since the 3PRs also face fewer demands, they will deliberately decrease the production quantity of the remanufactured products and the carbon emissions; thus, the 3PRs will sell their excess carbon emission allowances to other enterprises to obtain more profits.

**Figure 11:**
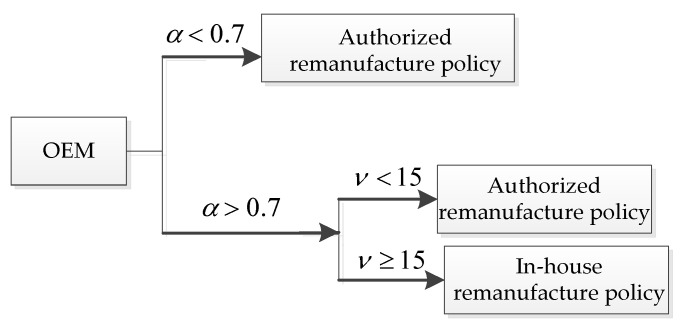
The effect of carbon trading system on OEMs’ remanufacturing strategies.

For different lower limits of the recovery rate α, the unit carbon trading prices ν have different effects on the OEMs’ choice of manufacturing decision. In other words, for each (α,ν), the OEMs will choose the remanufacturing policies that provide them with better profits. The decision diagram of the OEMs’ manufacturing policies based on different values of (α,ν) is shown in Figure 11.

Thus, the results of this section show that the establishment of a governmentally controlled carbon trading system will prompt companies to reduce their production of new products and increase their production of remanufactured products. From the perspective of the OEMs’ profits, the implementation of the carbon trading system will reduce the company’s profits to a certain extent. When the lower limit of the recovery rate is low, OEMs will choose the ARS. Otherwise, the OEMs will choose their remanufacturing strategies according to the unit carbon trading price as follows: for a lower unit carbon trading price, they would choose the ARS, otherwise, they would choose the IHRS. Thus, the implementation of the carbon trading system can reduce the amount of carbon emissions by forcing the enterprises to produce fewer products, thereby protecting the environment.

### 6.2. The Impact of the Green Innovation Efforts

This section mainly analyzes the impact of the green innovation efforts made by the OEMs on the sales quantities, product prices, profits of each decision-maker, and the OEMs’ remanufacturing strategy choices. Since the lower limit of the recovery rate of the used products can also affect the number of new products and remanufactured products in the demand markets, it can ultimately affect the choice of the OEMs’ remanufacturing strategy. Thus, we analyze the effect of the green innovation efforts on the decision variables and profits by using different lower limits of the recovering rates of the used products α: Case 1: α<0.5 (we use α=0.4 for illustrative purpose.); Case 2: α>0.5 (we use α=0.6 for illustrative purpose). In each of these cases, the other parameter settings are the same, i.e., β=0.9, $\bar{\rho }=0.5$, τ=0.5, δ=0.8, $e_{n}=2$, $e_{r}=1.5$, ν=10, and $\bar{M}=10$. We obtain the equilibrium of the decision-makers, the carbon emission costs, and the profit of each decision-maker in both the IHRS model and the ARS model corresponding to different green innovation χ, where χ varies between 0 and 1, to obtain the equilibrium decisions.

Case 1: Low lower limit of the recovery rate (α=0.4).

**Figure 12:**
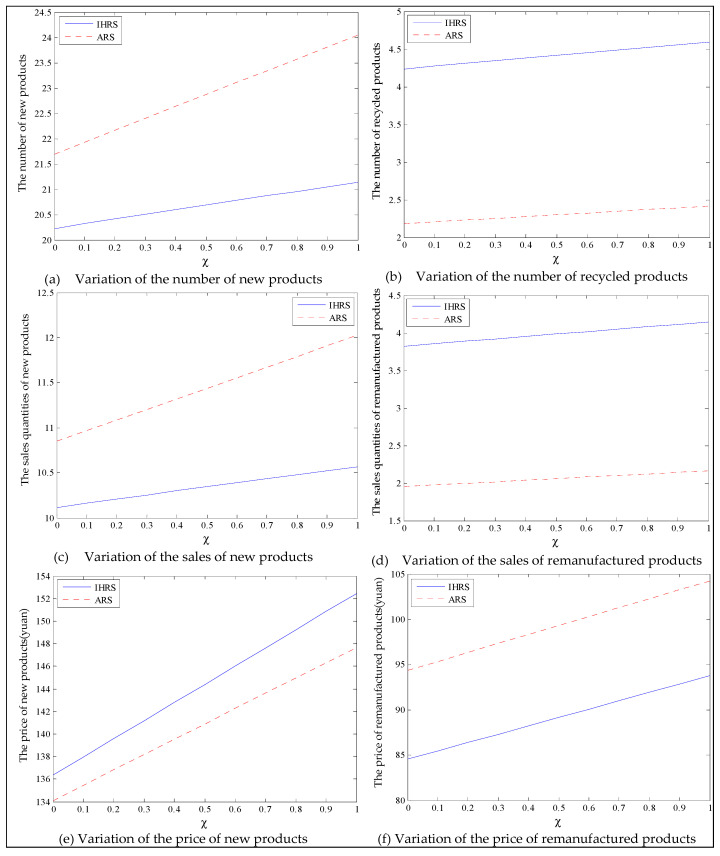
The variation of equilibrium strategies in Case 1 of Section 6.2.

**Figure 13:**
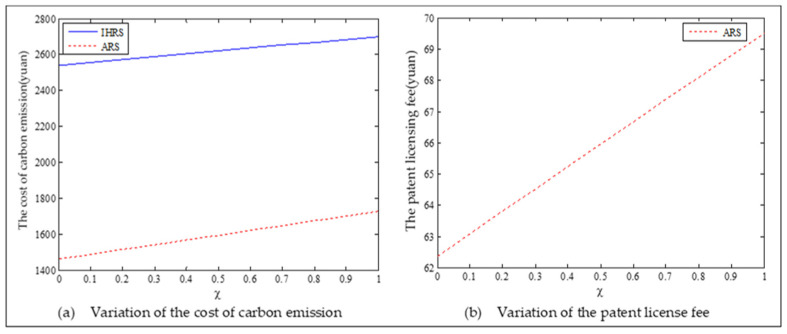
Changes of the costs of carbon emission and the patent licensing fee in Case 1 of Section 6.2.

**Figure 14:**
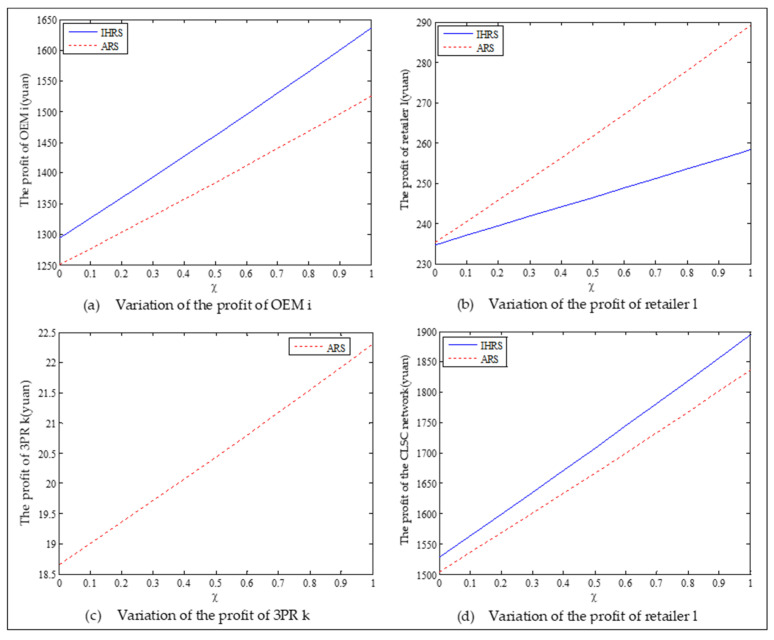
The variation of the profits in Case 1 of Section 6.2.

The equilibrium of the decision-makers is shown in Figure 12; the carbon emission costs due to the production of these two products of the IHRS model and the ARS model, and the patent licensing fee are shown in Figure 13; the profits of each decision-maker and that of the CLSC network are shown in Figure 14.

When the lower limit of the recovery rate is low, we observe from Figure 12 that when the green innovation efforts increase, the production quantities of the new products of both the IHRS model and the ARS model increase, but their recycling quantities decrease; the sales quantities of these two products of both the IHRS model and the ARS model increase and their prices also increase, but the increase in sales quantity of the remanufactured products is not as significant as that of the new products. The above directions of movement of the decision variables with respect to the green innovation efforts are consistent with the statements of Proposition 4 and Proposition 10. Moreover, for Case 1, when the OEMs choose the IHRS, the sales quantities of the new products are lower than when they choose the ARS, but their prices are higher than when they choose the ARS. On the other hand, for Case 1, when the OEMs choose the IHRS, the quantities of the recycling products and the sales of the remanufacturing products are higher than when they choose the ARS, but the prices of the remanufactured products are lower than when they choose the ARS. From Figure 13, when the green innovation efforts increase, the carbon emission costs charged to the OEMs and the 3PRs to produce these two products increase, but the carbon emission cost of the IHRS model is much higher than that of the ARS model; the patent licensing fees paid by the 3PRs to the OEMs also increase. From Figure 14, when the green innovation efforts increase, the profits of the OEMs, the retailers, the 3PRs, and the entire CLSC network increase; however, the profits of the 3PRs and the entire CLSC network of the IHRS model are higher than those of the ARS model.

For Case 1, when the green innovation efforts increase, fewer products are cycled for remanufacturing due to the low lower limit of the recovery rate. Thus, new products occupy most of the markets, and the demand of the consumers for new products increases. Due to the relationship between supply and demand, the OEMs will choose to increase the production of the new products, and the prices of the new products will increase. Although the green efforts of the OEMs will increase the production costs of the new products, the sales of the new products will generate more profits for the OEMs, which leads to an increase in profits for the entire supply chain. When the green innovation efforts increase, although the demands of the 3PRs will be less than those of the OEMs, the production costs of the remanufactured products will be reduced and the demands for the remanufactured products from the consumers will increase; thus, the profits of the 3PRs will also increase.

Case 2: High lower limit of the recovery rate (α=0.6).

**Figure 15:**
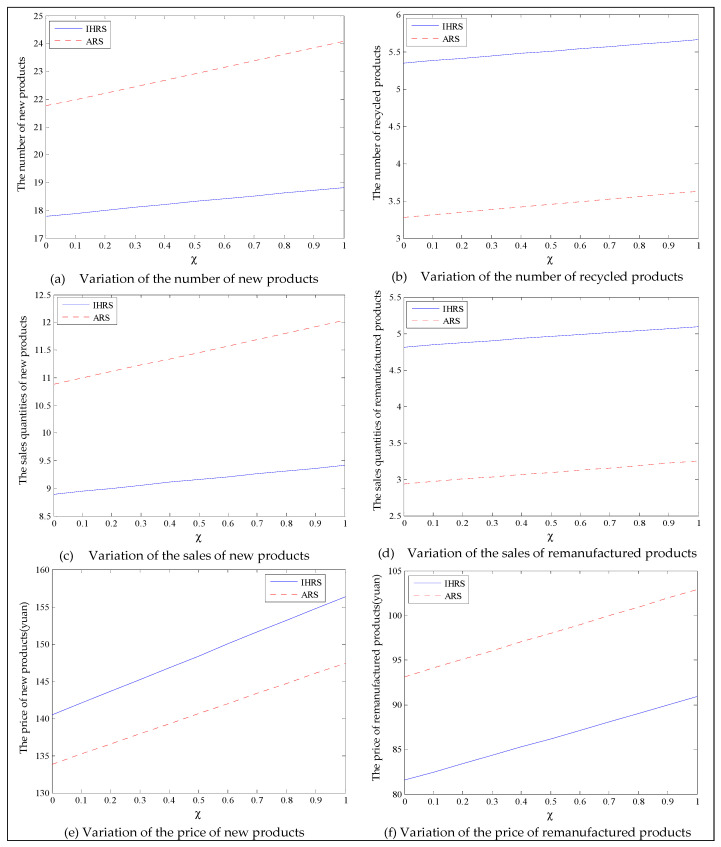
The variation of equilibrium strategies in Case 2 of Section 6.2.

**Figure 16:**
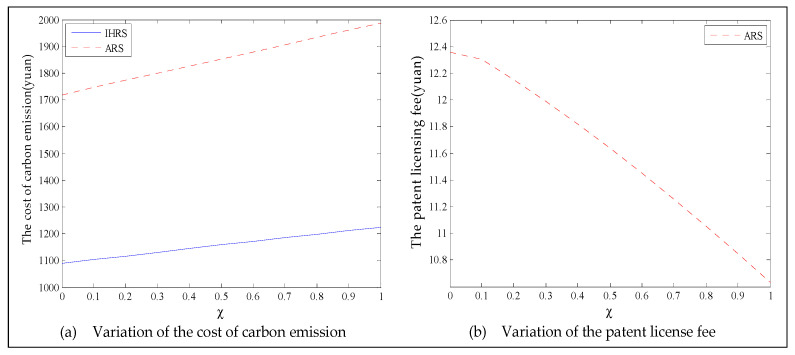
Changes of the costs of carbon emission and the patent licensing fee in Case 2 of Section 6.2.

**Figure 17:**
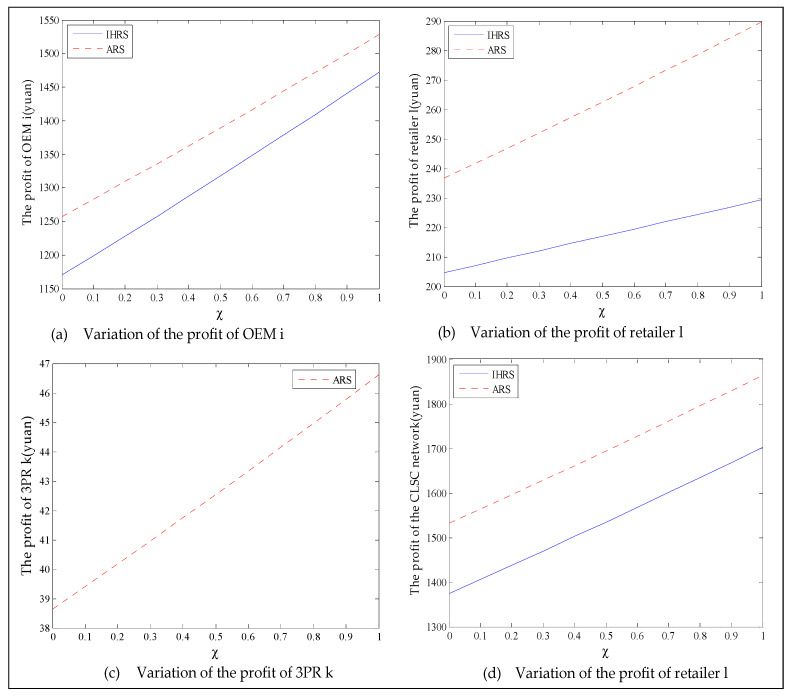
The variation of the profits in Case 2 of Section 6.2.

The equilibrium of the decision-makers is shown in Figure 15; the carbon emission costs due to the production of these two products of the IHRS model and the ARS model, and the patent licensing fee are shown in Figure 16; the profit of each decision-maker and that of the CLSC network is shown in Figure 17.

For Case 2, from Figure 15 and Figure 16, when the lower limit of the recycling increases, the differences in production quantity and sales quantity of each decision-maker between the IHRS model and the ARS model become significant; in other words, the production quantity and sales quantity of the new products of the ARS model are larger than those of the IHRS, whilst the recycling quantity and the production quantity of the remanufacturing products of the IHRS model are larger than those of the ARS model. The directions of movement for all the decision variables in both models with respect to the green innovation efforts are the same as those for Case 1, which is consistent with the statements of Proposition 4 and Proposition 10. Moreover, when the innovation efforts increase, the authorization cost paid by the 3PRs for the collection of used products tends to be 0. From Figure 17, the companies’ improvement of their green innovation efforts will increase the profits of the OEMs, the retailers, the 3PRs, and the entire supply chain; however, the profits of the OEMs when they choose the IHRS are larger than when they choose the ARS. When both the recycling rate of the new products and the green innovation efforts of the OEMs are extremely high, the high recycling rate increases the number of remanufactured products, and the high green innovation efforts of the OEMs also reduce the production costs; thus, the companies will focus more on the production of the remanufactured products than on new products, which provides the OEMs with better recycling and remanufacturing performances under the IHRS model; thus, the OEMs choose to remanufacture the used products themselves instead of authorizing the 3PRs to remanufacture the used products to obtain more profits.

**Figure 18:**
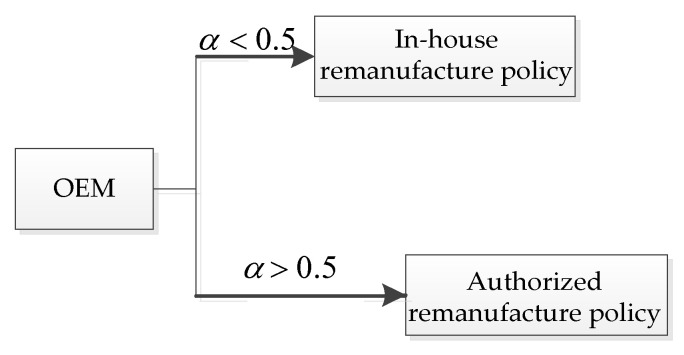
The effect of the green innovation efforts on OEMs’ remanufacturing strategies.

For different lower limits of the recovery rate α, the green innovation efforts χ have different effects on the OEMs’ choice of remanufacturing decision. In other words, for each (α,χ), the OEMs will choose the remanufacturing strategies that provide them with better profits. The decision diagram of the OEMs’ remanufacturing strategies based on different values of (α,χ) is shown in Figure 18.

From the results of this section, the outcomes of the green innovation efforts made by the OEMs during the production periods are positive. From the perspective of the OEMs’ profits, in both the IHRS model and the ARS model, the green innovation efforts of the OEMs will prompt the companies to increase the production and the sales of these two products, and hence help the companies increase their profits. The choice of OEMs is affected by both the lower limit of the recovery rate and the green innovation efforts as follows: the OEMs will choose the IHRS when the lower limit of the recovery rate is low and will choose the ARS when the lower limit of the recovery rate is high. From the perspective of carbon emissions, the ARS model produces fewer carbon emissions when the lower limit of the recovery rate is low, and the IHRS model is more environmentally friendly when the lower limit of the recovery rate is high.

### 6.3. The Impact of the Proportion of Green Consumers

This section mainly analyzes the impact of the proportion of green consumers on the sales quantities, the prices of the products, the profit of each decision-maker, and the OEMs’ choice of remanufacturing strategy. Since the lower limits of the recovering rates of the used products can also affect the number of new products and remanufactured products in the demand markets, it can ultimately affect the OEMs’ choice of remanufacturing strategy. Thus, we analyze the effect of the proportion of green consumers on the decision variables and profits by using different lower limits for the recovering rates of the used products: α: Case 1: α<0.6 (we use α=0.4 for illustrative purposes); Case 2: α>0.6 (we use α=0.7 for illustrative purposes). In each of these cases, the other parameter settings are the same, i.e., β=0.9, $\bar{\rho }=0.5$, ν=10, χ=0.5, δ=0.8, $e_{n}=2$, $e_{r}=1.5$, and $\bar{M}=10$. We obtain the equilibrium of the decision-makers, the carbon emission costs, and the profit of each decision-maker in both the IHRS model and the ARS model corresponding to the different proportion of green consumers τ, where τ varies between 0.1 and 0.9 to get the equilibrium decisions.

Case 1: Low lower limit of the recovery rate (α=0.4).

**Figure 19:**
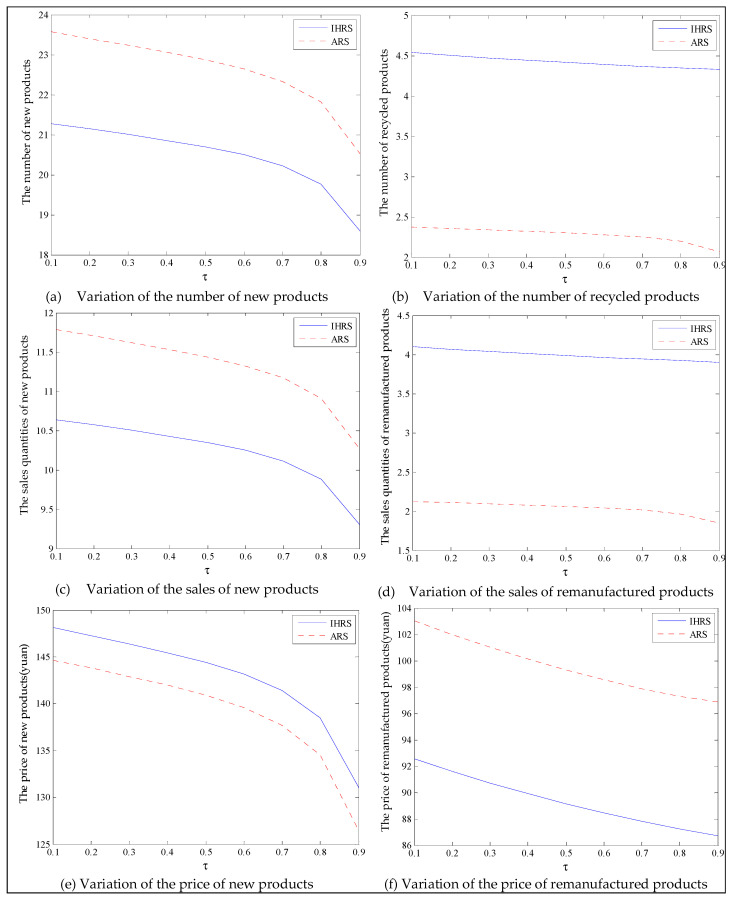
The variation of equilibrium strategies in Case 1 of Section 6.3.

**Figure 20:**
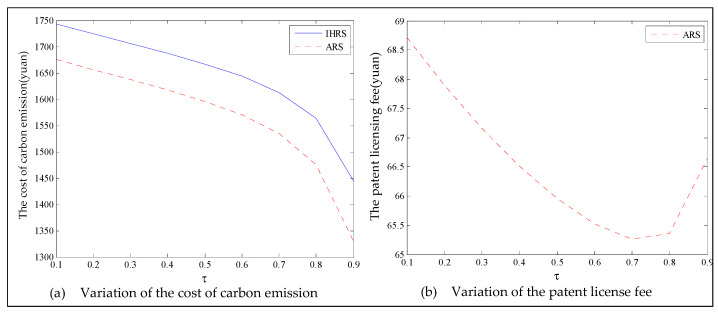
Changes in the costs of carbon emission and the patent licensing fee in Case 1 of Section 6.3.

**Figure 21:**
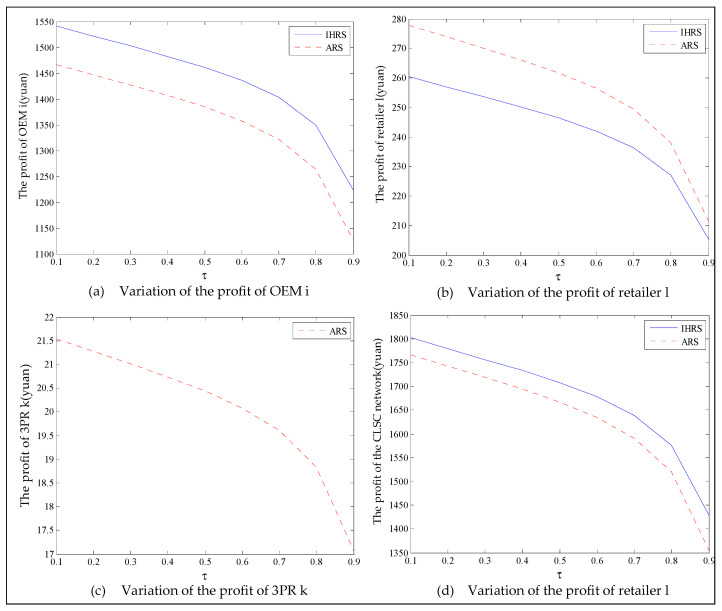
The variation of the profits in Case 1 of Section 6.3.

The equilibrium of the decision-makers is shown in Figure 19; the carbon emission costs due to the production of these two products of the IHRS model and the ARS model, and the patent licensing fee are shown in Figure 20; the profit of each decision-maker and the profit of the CLSC network are shown in Figure 21.

When the lower limit of the recovery rate is low, the emergence of green consumers leads to a downward trend in decision variables. We observe from Figure 19 that when the proportion of the green consumers increases, the production quantities, sales, and prices of the new products of both the IHRS model and the ARS model largely decrease; the recycling quantities and the sale quantities of the remanufactured products slightly decrease, but the sales quantity of the remanufactured products in the IHRS model is higher than that of the ARS model. The above directions of movement for the decision variables with respect to the proportion of green consumers are consistent with the statements of Proposition 5 and Proposition 11. From Figure 20, when the proportion of the green consumers τ increases from 0.1 to 0.9, the carbon emission costs charged to the OEMs and the 3PRs to produce these two products gradually decreases, but the carbon emission cost of the IHRS model is higher than of the ARS model. The patent licensing fees paid by the 3PRs to the OEMs first decrease and then increase, and the patent licensing fee paid by the 3PRs to the OEMs has its minimum value when τ=0.7. From Figure 21, when the proportion of the green consumers increases, the profits of the OEMs, the retailers, the 3PRs, and hence the entire CLSC network decrease. When the lower limit of the recycling rate is low, there are fewer products available for recycling and remanufacturing in the market, and the emergence of environmentally conscious green consumers further reduces the demand for these two products. Thus, to increase sales and obtain more profits, the OEMs and 3PRs will choose to decrease the selling price of their products. On the other hand, OEMs will consider charging 3PRs with a higher patent licensing fee to compensate the loss of profits due to the decrease in sales quantities.

Case 2: High lower limit of the recovery rate (α=0.7).

**Figure 22:**
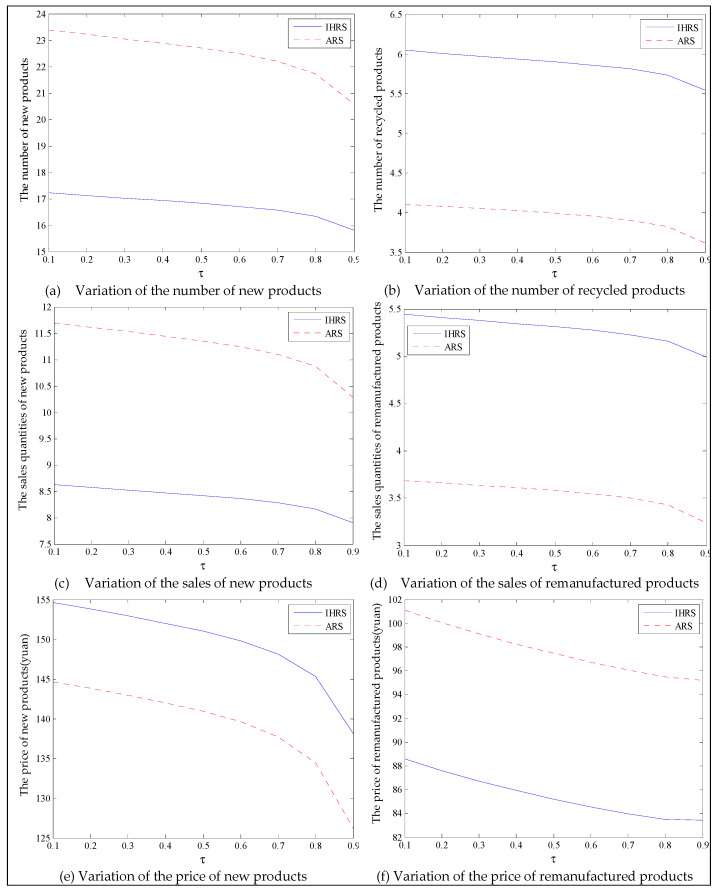
The variation of equilibrium strategies in Case 2 of Section 6.3.

**Figure 23:**
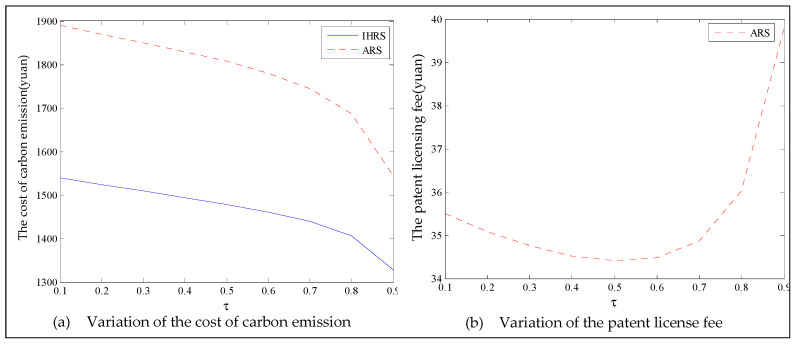
Changes in the carbon emission costs and patent licensing fee in Case 2 of Section 6.3.

**Figure 24:**
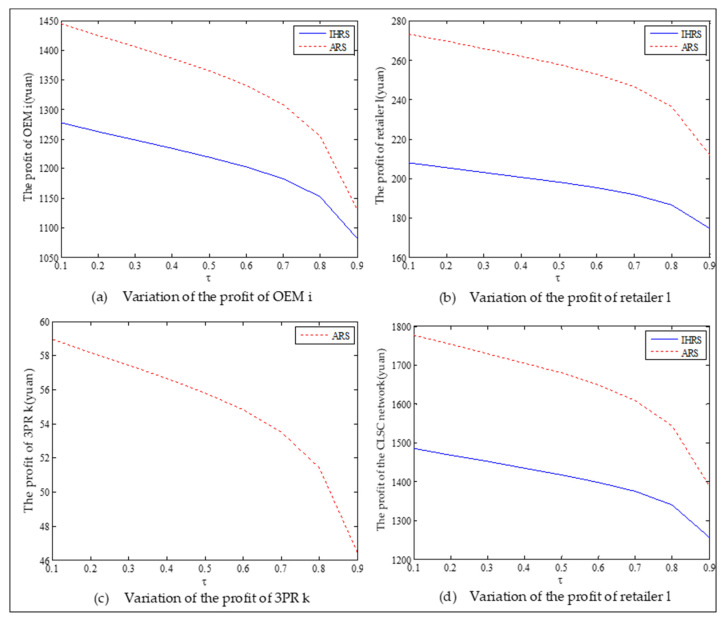
The variation of the profits in Case 2 of Section 6.3.

The equilibrium of the decision-makers is shown in Figure 22; the carbon emission costs due to the production of these two products of the IHRS model and the ARS model, and the patent licensing fee are shown in Figure 23; the profit of each decision-maker and the profit of the CLSC network are shown in Figure 24.

When the lower limit of the recycling rate is high, from Figure 22, the directions of movement for all the decision variables in both models with respect to the proportion of the green consumers in the markets are the same as those for Case 1, which is consistent with the statements of Proposition 5 and Proposition 11. For Case 2 (high lower limit of the recovery rate), from Figure 22, Figure 23 and Figure 24, when the lower limit of the recycling increases, the differences in the production quantity and the sales quantity of the new products between the IHRS model and the ARS model become significant; the profits of the OEMs and the retailers between the IHRS model and the ARS model also become more significant; when the proportion of the green consumers’ efforts τ increases from τ=0.1 to τ=0.5, the patent licensing fee charged to the 3PRs first decreases, and when the proportion of the green consumers’ efforts increases from τ=0.5 to τ=0.9, the patent licensing fee charged to the 3PRs sharply increases.

When the lower limit of the recovery rate is high, the company may produce products that do not meet the standards or expectations of consumers to reach the lower limit of the specified recovery rate. Thus, some consumers refuse to buy such products, which leads to decreases of the sales quantity for these two products. When the proportion of green consumers is low, the licensing fees charged to the 3PRs decrease due to the decrease in sales quantities of the remanufactured products. However, when the proportion of green consumers is high, the sales of these two products drop significantly; thus, the OEMs must charge the 3PRs with higher patent licensing fees to compensate for the loss of profits. Therefore, the lower limit of the recovery rate should be set according to the situation of the markets, as an excessively high recovery rate will sharply reduce the quality of the products produced by the enterprise.

**Figure 25:**
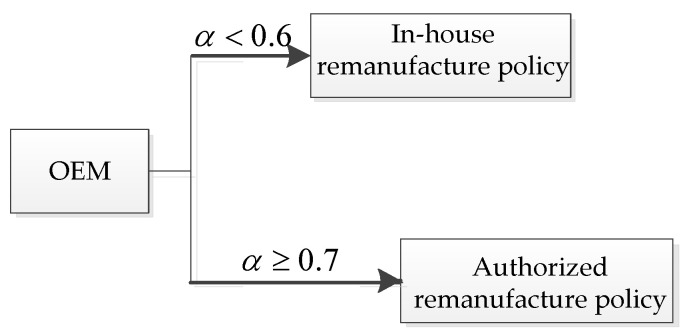
The effect of the green consumers on OEMs’ remanufacturing strategies.

For different lower limits of the recovery rate α, the proportion of green consumers τ has different effects on the OEMs’ choice of remanufacturing decision. In other words, for each (α,τ), the OEMs will choose the remanufacturing strategies that provide them with better profits. The decision diagram of the OEMs’ remanufacturing strategies based on different values of (α,τ) is shown in Figure 25.

Thus, from the results of this section, the emergence of green consumers reduces the production and the prices of these two products, resulting in a slight reduction in the profits of the decision-makers. However, on the other hand, the emergence of green consumers reduces the quantity of carbon emissions. From the perspective of the OEMs’ profit, when the lower limit of the recovery rate is less than 0.6, the OEMs will choose the IHRS; when the lower limit of the recovery rate is higher than 0.6, the OEMs will choose the ARS to obtain the maximum profit. From the perspective of carbon emissions, the increase in the green consumers will greatly reduce the amount of carbon emission in the two models; when the lower limit of the recovery rate is low, the amount of carbon emission due to the ARS model is less than that due to the IHRS model, but when the lower limit of the recovery rate is higher, the situation is reversed. Therefore, although the emergence of green consumers reduces the profits of the decision-makers, it indirectly promotes green behaviors among the enterprises. While reducing the profits of the enterprise, the emergence of green consumers can effectively reduce carbon emissions and bring long-term environmental benefits, which can help the long-term development of the enterprises.

## 7. Conclusions

Due to the increasingly serious environmental pollution problems and governments’ strengthening of environmental impact supervision, manufacturing companies are seeking green production methods, implementing carbon trading systems, and promoting the trend toward green remanufacturing. Thus, this paper introduces green factors, namely carbon trading, green innovation efforts, and green consumers, to the existing CLSC network models and studies the impacts of these factors on the choice between two remanufacturing strategies: IHRS and ARS. Using VI, we obtain the optimal strategies of the OEMs, the retailers, the 3PRs, and the CLSC network, together with their associated economic analyses. These economic analyses are not only beneficial to manufacturing enterprises for exploring more resource-saving and greener production methods, but they are also beneficial to the government for the implementation of environmental protection strategies. The conclusions are as follows:

1. The impacts of these green factors are different, the increase of the carbon trading price and the proportion of green consumers will decrease the production and sales quantities of new products, and the recycling quantities of the used product. However, the movement of these decision variables with respect to the green innovation efforts are in the opposite directions;
2. From the perspective of obtaining greater profits, the choice of the OEMs in the network between two remanufacturing strategies is affected by the green factors and the recovery rate of the used product. We observe from Figure 11 that when the carbon trading price is low, the OEMs choose the ARS; when the carbon trading price is high, the OEMs choose the IHRS. From Figure 18 and Figure 25, for all the green innovation effort levels and the proportions of green consumers, when the recovery rate of the used product is low, the OEMs choose the IHRS; when the recovery rate of the used product is high, the OEMs choose the ARS;
3. From the perspective of protecting the environment and reducing environmental pollution, the OEMs choose the ARS when the values of the green factors are low; the OEMs choose the IHRS when the values of the green factors are high;
4. The unit carbon trading price set by the government should be appropriate because a price that is too low will cause companies to ignore the existence of the system, while a price that is too high will cause the companies to lose enthusiasm for the production of their products. Therefore, the governments should establish the appropriate unit carbon trading price only after they have thoroughly investigated the market situation, which is consistent with the viewpoint of [33].

However, this paper still has some limitations. This paper only studies the comparison of the performance between the IHRS and the ARS of the OEMs in the CLSC network, but it does not consider other remanufacturing strategies, such as the outsourcing remanufacturing and the retailer remanufacturing strategies. In the future, we shall incorporate these strategies into the CLSC network so that we can compare the performance of each of the above strategies. Moreover, this paper assumes that all decision-makers are completely rational, but in real life, the investors of enterprises are not completely rational [48]. Thus, in the future, we shall also analyze the optimal strategies in the CLSC network from the perspective of behavioral economics.

## Figures and Tables

**Table 1:** The characteristics of this paper.

Reference	CLSC Network		In-House Remanufacturing		Authorized Remanufacturing		Carbon Emission		Green Innovation		Green Consumers	
	Yes	No	Yes	No	Yes	No	Yes	No	Yes	No	Yes	No
Majumder and Groenevelt. (2001) [18]	√			√	√			√		√		√
Qiang. (2015) [13]	√			√		√		√		√		√
Chai et al. (2018) [26]		√		√		√	√			√	√	
Fu et al. (2021) [23]	√			√		√		√		√		√
Lv and Li. (2021) [33]		√		√		√		√	√		√	
Wang et al. (2021) [34]		√	√			√	√			√		√
Zhou et al. (2021) [14]	√			√		√		√		√		√
Zhou et al. (2021) [40]	√		√		√			√		√		√
Cheng et al. (2022) [35]	√		√			√	√			√	√	
Yang et al. (2022) [38]	√		√			√	√			√		√
This study	√		√		√		√		√		√	

The “√” represents “yes”.

**Table 2:** The decision variables in the IHRS model.

Symbol	Definition
$q_{i}^{v}$	The non-negative number of new products produced by OEM i. All new products produced by all the OEMs form a vector $Q^{v}\in R_{+}^{M}$.
$q_{i,l}^{n}$	The non-negative sale of new products between OEM i and retailer l. All sales of new products form a matrix $Q^{1}\in R_{+}^{MT}$.
$q_{i,l}^{r}$	The non-negative sale of remanufactured products between OEM i and retailer l. All sales of remanufactured products form a matrix $Q^{2}\in R_{+}^{MT}$.
$q_{l,j}^{n}$	The non-negative sale of new products between retailer l and market j. All sales of new products form a matrix $Q^{3}\in R_{+}^{TN}$.
$q_{l,j}^{r}$	The non-negative sale of remanufactured products between retailer l and market j. All sales of remanufactured products sold by all retailers to all demand markets form a matrix $Q^{4}\in R_{+}^{TN}$.
$q_{j,i}^{e}$	The non-negative number of used products returned to OEM i from demand market j. The numbers of used products returned to all OEMs from all demand markets form a matrix $Q^{5}\in R_{+}^{NM}$.
$\rho_{j}^{n}$	The price for purchasing 1 item of new product at demand market j. The prices for purchasing 1 item of new product at all demand markets form a vector $\rho^{n}\in R_{+}^{N}$ (unit: yuan/item).
$\rho_{j}^{r}$	The prices for purchasing 1 item of remanufactured product at all demand markets form a vector $\rho^{r}\in R_{+}^{N}$ (unit: yuan/item).

**Table 3:** The parameters in the IHRS model.

Symbol	Definition
α	The lower limit for the percentage of used products returned from demand markets.
β	The fraction of used products that can be used for remanufacturing $\left(\bar{\beta }=1-\beta \right)$.
$\bar{\rho }$	The cost for sending 1 item of waste product to the landfill for disposal.
ν	The unit price of carbon trading fixed by the Government.
χ	The green innovation efforts of OEMs.
τ	The percentage of green consumers in the market.
δ	The consumers’ discount on purchasing remanufactured products over purchasing new products.
$e_{n}$	The environmental impact per item of the new products. (Unit: kg/item).
$e_{r}$	The environmental impact per item of the remanufactured products. (Unit: kg/item).
$\bar{M}$	The upper limit of carbon emission during one production period imposed by the Government.

**Table 4:** The cost functions in the IHRS model.

Symbol	Definition
$f_{i}\left(Q^{v},\chi \right)$	The production cost of the new products of OEM i (Unit: yuan).
$c_{i,l}^{n}\left(q_{i,l}^{n}\right)$	The transaction cost of $q_{i,l}^{n}$ items of the new products between OEM i and retailer l (Unit: yuan).
$c_{i,l}^{r}\left(q_{i,l}^{r}\right)$	The transaction cost of $q_{i,l}^{r}$ items of the remanufactured products between OEM i and retailer l. (Unit: yuan).
$c_{l}^{n}\left(Q^{1}\right)$	The total exhibition cost of new products of retailer l. (Unit: yuan).
$c_{l}^{r}\left(Q^{2}\right)$	The total exhibition cost of remanufactured products for the retailer l. (Unit: yuan).
$\phi_{i}\left(\sum_{j=1}^{N}q_{j,i}^{e}\right)$	The total inspection cost of OEM i for the collection of all used products. (Unit: yuan).
$c_{j,i}^{e}\left(q_{j,i}^{e}\right)$	The transaction cost of OEM i for the purchase of $q_{j,i}^{e}$ items of used products from demand market j. (Unit: yuan).
$c_{i,D}\left(\bar{\beta }\sum_{j=1}^{N}q_{j,i}^{e}\right)$	The total transportation cost of OEM i for transferring the waste products from all the demand markets to the landfill for disposal. (Unit: yuan).
$r_{i}\left(\sum_{l=1}^{T}q_{i,l}^{r},\chi \right)$	The total production cost of OEM i for the remanufacturing of returned products from all the retailers. (Unit: yuan).
$a_{j}\left(\sum_{i=1}^{M}q_{j,i}^{e}\right)$	The total collection cost of demand market j for returning used products to all the OEM. (Unit: yuan).
$c_{l,j}^{n}\left(q_{l,j}^{n}\right)$	The transaction cost of $q_{l,j}^{n}$ items of the new products between retailer l and market j (Unit: yuan).
$c_{l,j}^{r}\left(q_{l,j}^{r}\right)$	The transaction cost of $q_{l,j}^{r}$ items of the remanufactured products between retailer l and market j (Unit: yuan).

$f_{i}\left(Q^{v},\chi \right)$ is a function of both the number of new products manufactured by OEM i and the green innovation effort of the OEMs; $r_{i}\left(\sum_{l=1}^{T}q_{i,l}^{r},\chi \right)$ is a function of both the number of remanufactured products and the green innovation effort.

**Table 5:** The decision variables, parameters, and cost functions in the ARS model.

Symbol	Definition
$q_{j,k}^{e}$	The non-negative number of used products returned to 3PR k from demand market j. All used products form a matrix $Q^{6}\in R_{+}^{NO}$.
$q_{k,i}^{r}$	The non-negative sale of remanufactured products transacted between OEM i and 3PR k. All remanufactured products form a matrix $Q^{7}\in R_{+}^{OM}$.
$q_{k,j}^{r}$	The non-negative sale of new products between 3PR k and market j. All remanufactured products form a matrix $Q^{8}\in R_{+}^{ON}$.
ϑ	The remanufacturing ratio fixed by the OEMs.
$a_{j}\left(\sum_{k=1}^{O}q_{j,k}^{e}\right)$	The total cost of demand market j for returning used products to all the 3PRs. (Unit: yuan).
$c_{j,k}^{e}\left(q_{j,k}^{e}\right)$	The transaction cost of 3PR k for the collection of $q_{j,k}^{e}$ items of used products. (Unit: yuan).
$c_{k,j}^{r}\left(q_{k,j}^{r}\right)$	The remanufacturing cost of 3PR k for the remanufacturing of $q_{k,j}^{r}$ items of remanufactured products. (Unit: yuan).
$\phi_{k}\left(\sum_{j=1}^{N}q_{j,k}^{e}\right)$	The total inspection cost of 3PR k for the collection of all used products. (Unit: yuan).
$r_{k}\left(\sum_{i=1}^{M}q_{k,i}^{r},\chi \right)$	The total production cost of 3PR k for the remanufacturing of remanufactured products from all the OEMs.
$c_{k,D}\left(\bar{\beta }\sum_{j=1}^{N}q_{j,k}^{e}\right)$	The total transportation cost of 3PR k for transferring the waste products from all the demand markets to the landfill for disposal. (Unit: yuan).

## Appendix A

For the sake of simplicity, we denote the profit function $\prod_{i}^{1}$ in Equation (3) by f and its Hessian matrix by H. Then, by differentiating f with respect to the decision variables $q_{i}^{v},q_{i,l}^{n},q_{i,l}^{r},$ and $q_{j,i}^{e}$, we obtain the gradient vector

(A1) $$\nabla f=\left[\begin{array}{c} -\frac{\partial f_{i}\left(Q^{v},\chi \right)}{\partial q_{i}^{v}}-ve_{n}\\ p_{i,l}^{n}-\frac{\partial c_{{}_{i,l}}^{n}\left(q_{i,l}^{n}\right)}{\partial q_{i,l}^{n}}\\ p_{i,l}^{r}-\frac{\partial r_{i}\left(q_{i,l}^{r},\chi \right)}{\partial q_{i,l}^{r}}-\frac{\partial c_{{}_{i,l}}^{r}\left(q_{i,l}^{r}\right)}{\partial q_{i,l}^{r}}-ve_{r}\\ \rho_{j}^{e}+\frac{\partial c_{j,i}^{e}\left(q_{j,i}^{e}\right)}{\partial q_{j,i}^{e}}-\frac{\partial \phi_{i}\left(q_{j,i}^{e}\right)}{\partial q_{j,i}^{e}}-\bar{\beta }\bar{\rho }-\frac{\partial c_{i,D}\left(\bar{\beta }q_{j,i}^{e}\right)}{\partial q_{j,i}^{e}} \end{array}\right]$$

Hence, we get the Hessian matrix

(A2) $$H=\left[\begin{array}{cccc} -\frac{\partial^{2}f_{i}}{\partial q_{i}^{v}{}^{2}} & 0 & 0 & 0\\ 0 & -\frac{\partial^{2}c_{i,l}^{n}}{\partial q_{i,l}^{n}{}^{2}} & 0 & 0\\ 0 & 0 & -\frac{\partial^{2}r_{i}}{\partial q_{i,l}^{r}{}^{2}}-\frac{\partial^{2}c_{i,l}^{r}}{\partial q_{i,l}^{r}{}^{2}} & 0\\ 0 & 0 & 0 & -\frac{\partial^{2}c_{j,i}^{e}}{\partial q_{j,i}^{e}{}^{2}}-\frac{\partial^{2}\phi_{i}}{\partial q_{j,i}^{e}{}^{2}}-\frac{\partial^{2}c_{i,D}}{\partial q_{j,i}^{e}{}^{2}} \end{array}\right]$$

From Assumption 7, we know that all the minors of H with odd dimensions of H are less than 0, and all the minors of H with even dimensions are greater than 0. Thus, the Hessian matrix H is negative definite. Thus, the profit Function (3) is concave with respect to its decision variables.

## Appendix B

The Lagrange function of the optimization problem defined by (3)–(6) is

(A3) $$\begin{array}{l} L_{i}=-\sum_{l=1}^{T}p_{i,l}^{n}q_{i,l}^{n}-\sum_{l=1}^{T}p_{i,l}^{r}q_{i,l}^{r}+f_{i}\left(Q^{v},\chi \right)+r_{i}\left(q_{i,l}^{r},\chi \right)+\rho_{j}^{e}q_{j,i}^{e}+c_{j,i}^{e}\left(q_{j,i}^{e}\right)+\phi_{i}\left(q_{j,i}^{e}\right)+\\ \bar{\beta }\bar{\rho }q_{j,i}^{e}+c_{i,D}\left(\bar{\beta }q_{j,i}^{e}\right)+\sum_{l=1}^{T}c_{{}_{i,l}}^{n}\left(q_{i,l}^{n}\right)+\sum_{l=1}^{T}c_{{}_{i,l}}^{r}\left(q_{i,l}^{r}\right)+v\left(e_{n}q_{i}^{v}-M_{m}\right)+v\left(e_{r}\sum_{l=1}^{T}q_{i,l}^{r}-M_{m}\right)+\\ \varepsilon_{i}\left(q_{i}^{v}-\sum_{l=1}^{T}q_{i,l}^{n}\right)+\mu_{i}\left(\sum_{j=1}^{N}q_{j,i}^{e}-\alpha \sum_{l=1}^{T}q_{i,l}^{n}\right)+\varphi_{i}\left(\beta \sum_{j=1}^{N}q_{j,i}^{e}-\sum_{l=1}^{T}q_{i,l}^{r}\right) \end{array}$$

By differentiating $L_{i}$ with respect to each of the decision variables $q_{i}^{v},q_{i,l}^{n},q_{i,l}^{r},q_{j,i}^{E},\varepsilon_{i},\mu_{i},$ and $\varphi_{i}$, we obtain

(A4) $$\frac{\partial L_{i}}{\partial q_{i}^{v}}=\frac{\partial f_{i}\left(Q^{v}\right)}{\partial q_{i}^{v}}-\varepsilon_{i}^{}+ve_{n}$$

(A5) $$\frac{\partial L_{i}}{\partial q_{i,l}^{n}}=-p_{i,l}^{n}+\frac{\partial c_{i,l}^{n}\left(q_{i,l}^{n*}\right)}{\partial q_{i,l}^{n}}+\alpha \mu_{i}^{}+\varepsilon_{i}^{}$$

(A6) $$\frac{\partial L_{i}}{\partial q_{i,l}^{r}}=-p_{i,l}^{r}+\frac{\partial r_{i}\left(q_{i,l}^{r*},\chi \right)}{\partial q_{i,l}^{r}}+\frac{\partial c_{{}_{i,l}}^{r}\left(q_{i,l}^{r}\right)}{\partial q_{i,l}^{r}}+\varphi_{i}^{}+ve_{r}$$

(A7) $$\frac{\partial L_{i}}{\partial q_{j,i}^{e}}=\rho_{j}^{e}+\frac{\partial c_{j,i}^{e}\left(q_{j,i}^{e*}\right)}{\partial q_{j,i}^{e}}+\frac{\partial \phi_{i}\left(q_{j,i}^{e*}\right)}{\partial q_{j,i}^{e}}+\frac{\partial c_{i,D}\left(\bar{\beta }q_{j,i}^{e*}\right)}{\partial q_{j,i}^{e}}+\bar{\beta }\bar{\rho }-\mu_{i}^{}-\beta \varphi_{i}$$

(A8) $$\frac{\partial L_{i}}{\partial \varepsilon_{i}}=q_{i}^{v}-\sum_{l=1}^{t}q_{i,l}^{n}$$

(A9) $$\frac{\partial L_{i}}{\partial \mu_{i}}=\sum_{j=1}^{n}q_{j,i}^{e}-\alpha \sum_{l=1}^{t}q_{i,l}^{n}$$

(A10) $$\frac{\partial L_{i}}{\partial \varphi_{i}}=\beta \sum_{j=1}^{n}q_{j,i}^{e}-\sum_{l=1}^{t}q_{i,l}^{r}$$

## Appendix C

For the IHRS model, we use (A1) to obtain:

(A11) $$\frac{dq_{i}^{v}}{d\nu }=-\frac{e_{n}+\frac{\partial^{2}f_{i}\left(Q^{v},\chi \right)}{\partial q_{i}^{v}\partial \nu }}{\frac{\partial^{2}f_{i}\left(Q^{v},\chi \right)}{\partial q_{i}^{v}{}^{2}}}$$

(A12) $$\frac{dq_{i,l}^{n}}{d\nu }=-\frac{\frac{\partial^{2}c_{i,l}^{n}\left(q_{i,l}^{n}\right)}{\partial q_{i,l}^{n}\partial \nu }}{\frac{\partial^{2}c_{i,l}^{n}\left(q_{i,l}^{n}\right)}{\partial q_{i,l}^{n}{}^{2}}}$$

(A13) $$\frac{dq_{i,l}^{r}}{d\nu }=-\frac{\frac{\partial^{2}r_{i}\left(q_{i,l}^{r},\chi \right)}{\partial q_{i,l}^{r}\partial \nu }+\frac{\partial^{2}c_{i,l}^{r}\left(q_{i,l}^{r}\right)}{\partial q_{i,l}^{r}\partial \nu }+e_{r}}{\frac{\partial^{2}r_{i}\left(q_{i,l}^{r},\chi \right)}{\partial q_{i,l}^{r}{}^{2}}+\frac{\partial^{2}c_{i,l}^{r}\left(q_{i,l}^{r}\right)}{\partial q_{i,l}^{r}{}^{2}}}$$

(A14) $$\frac{dq_{j,i}^{e}}{d\nu }=-\frac{\frac{\partial^{2}c_{j,i}^{e}\left(q_{j,i}^{e}\right)}{\partial q_{j,i}^{e}\partial \nu }+\frac{\partial^{2}\phi_{i}\left(q_{j,i}^{e}\right)}{\partial q_{j,i}^{e}\partial \nu }+\frac{\partial^{2}c_{i,D}\left(\bar{\beta }q_{i,j}^{e}\right)}{\partial q_{i,j}^{e}\partial \nu }}{\frac{\partial^{2}c_{j,i}^{e}\left(q_{j,i}^{e}\right)}{\partial q_{j,i}^{e}{}^{2}}+\frac{\partial^{2}\phi_{i}\left(q_{j,i}^{e}\right)}{\partial q_{j,i}^{e}{}^{2}}+\frac{\partial^{2}c_{i,D}\left(\bar{\beta }q_{i,j}^{e}\right)}{\partial q_{i,j}^{e}{}^{2}}}$$

From the assumption, we know that $\frac{\partial^{2}f_{i}\left(Q^{v},\chi \right)}{\partial q_{i}^{v}\partial \nu }>0$, thus, by assuming that all the parameters and the sales quantities are larger than 0, we get $\frac{\partial q_{i}^{v}}{\partial \nu }<0$, $\frac{\partial q_{i,l}^{n}}{\partial \nu }<0$, $\frac{\partial q_{i,l}^{r}}{\partial \nu }<0$, and $\frac{\partial q_{j,i}^{e}}{\partial \nu }<0$.

Similarly, for the ARS model, we also obtain $\frac{\partial q_{i}^{v}}{\partial \nu }<0$, $\frac{\partial q_{i,l}^{n}}{\partial \nu }<0$, $\frac{\partial q_{j,k}^{e}}{\partial \nu }<0$, and $\frac{\partial q_{j,i}^{r}}{\partial \nu }<0$.

## Appendix D

From (1) and (2), we obtain $\frac{\partial d_{j}^{n}}{\partial \tau }<0$ and $\frac{\partial d_{j}^{r}}{\partial \tau }<0$. Since the production of the new products in the perfect market are completely determined by the consumers’ demand, we obtain $\frac{\partial q_{i}^{v}}{\partial \tau }<0$. Using similar arguments, $\frac{\partial q_{i,l}^{n}}{\partial \tau }<0$, $\frac{\partial q_{i,l}^{r}}{\partial \tau }<0$, and $\frac{\partial q_{j,i}^{e}}{\partial \tau }<0$.
